# Floods and Diarrhea Risk in Young Children in Low- and Middle-Income Countries

**DOI:** 10.1001/jamapediatrics.2023.3964

**Published:** 2023-10-02

**Authors:** Pin Wang, Ernest O. Asare, Virginia E. Pitzer, Robert Dubrow, Kai Chen

**Affiliations:** 1 Department of Environmental Health Sciences, Yale School of Public Health, New Haven, Connecticut; 2 Yale Center on Climate Change and Health, Yale School of Public Health, New Haven, Connecticut; 3 Department of Epidemiology of Microbial Diseases and the Public Health Modeling Unit, Yale School of Public Health, New Haven, Connecticut

## Abstract

**Importance:**

Climate change is associated with more frequent and intense floods. Current research on the association between flood exposure and diarrhea risk is limited mainly to short-term and event-specific analyses. Moreover, how prior drought or water, sanitation, and hygiene (WaSH) practices influence this association remains largely unknown.

**Objective:**

To examine the association between flood exposure and diarrhea risk among children younger than 5 years and to evaluate the compounding influence of prior drought and effect modification by WaSH.

**Design, Setting, and Participants:**

This cross-sectional study included multicluster surveys conducted by the Demographic and Health Surveys Program in 43 low- and middle-income countries during 2009 through 2019. This study included children younger than 5 years in all households from each survey cluster. Collected data were analyzed between September 1 and December 31, 2022.

**Exposures:**

Historical flood events during 2009 through 2019 were obtained from the Dartmouth Flood Observatory.

**Main Outcome and Measures:**

The main outcome was diarrhea prevalence among children younger than 5 years in the 2 weeks before the survey was conducted. Results were analyzed by binomial generalized linear mixed-effects logistic regression models with nested random intercepts for country and survey cluster.

**Results:**

Among 639 250 children making up the complete data series (excluding 274 847 children with missing values for diarrhea or baseline characteristics), 6365 (mean [SD] age, 28.9 [17.2] months; 3214 boys [50.5%]; 3151 girls [49.5%]) were exposed to floods during the 8 weeks after a flood started. The prevalence of diarrhea was 13.2% (n = 839) among exposed children and 12.7% (n = 80 337) among unexposed children. Exposure to floods was associated with increased diarrhea risk, with the highest odds ratio (OR) observed during the second to fourth weeks after floods started (OR, 1.35; 95% CI, 1.05-1.73). When floods were stratified by severity and duration, significant associations were observed only for extreme floods (OR during the third to fifth weeks, 2.07; 95% CI, 1.37-3.11) or floods lasting more than 2 weeks (OR during the second to fourth weeks, 1.47; 95% CI, 1.13-1.92), with significantly stronger associations than for less extreme floods or shorter-duration floods, respectively. The OR during the first 4 weeks after the start of floods was significantly higher for floods preceded by a 6-month or longer drought (12-month drought OR, 1.96; 95% CI, 1.53-2.52) than for floods not preceded by a 6-month or longer drought (12-month drought OR, 1.00; 95% CI, 0.79-1.27).

**Conclusions:**

These findings suggest that floods, especially severe floods, long-duration floods, and floods preceded by drought, are associated with an increased risk of diarrhea among children younger than 5 years living in low- and middle-income countries. With the projected increasing frequency and intensity of floods and drought under climate change, greater collective efforts are needed to protect children’s health from these compounding events.

## Key Points

**Question:**

Is there an association between flood exposure and diarrhea risk among children younger than 5 years living in low- and middle-income countries?

**Findings:**

In this multicountry cross-sectional study of 639 250 children, exposure to floods was found to be associated with a higher prevalence of diarrhea, driven by extreme flood events, floods that lasted more than 2 weeks, and floods preceded by droughts.

**Meaning:**

These findings suggest that more effective measures are warranted to protect children’s health from compound extreme weather events, including floods and drought, which are projected to increase in frequency and intensity due to climate change.

## Teaser

This cross-sectional study examines the association between flood exposure and diarrhea risk among young children in low- and middle-income countries, and evaluates whether water, sanitation, and hygiene practices have a role in this association.

## Introduction

Diarrhea remains one of the leading causes of death among children younger than 5 years,^1^ although its resultant mortality has decreased from approximately 1.6 million in 1990 to just more than half a million in 2019.^2^ Among the estimated 1.6 million total deaths (all ages) in 2016, almost 90% occurred in South Asia and sub-Saharan Africa.^3^

Diarrhea risk has been found to be associated not only with meteorologic parameters, such as rainfall,^4,5^ temperature,^4,6^ and humidity,^7^ but also with extreme weather events, such as cyclones or hurricanes,^8,9^ droughts,^10^ and floods.^4,5,9,11,12^ To our knowledge, all previous investigations on the association between floods and diarrhea risk have used data from single or multiple flood events during a short span of consecutive months or years in a single location.^4,5^ Results of these studies have been inconsistent,^4,5^ and the pooled relative risk from a recent meta-analysis was not statistically significant.^5^ In addition, although extreme precipitation following a dry period has been found to be associated with an increased risk of diarrhea,^5,13,14^ the dry conditions were simply defined as a low level of rainfall; no previous study has explored the combined effect of rigorously defined drought and flood on diarrhea risk.

Climate change leads to increased frequency and intensity of floods and droughts. Projected increases in global exposure to floods depend on the greenhouse gas emissions scenario, which will determine the increase in both temperature and extreme precipitation events,^15,16^ 2 main drivers of flood events.^15^

Inaccessibility to safe water, sanitation, and hygiene (WaSH) is a major risk factor for diarrhea that disproportionately affects populations with low socioeconomic status.^17^ It was estimated that 842 000 deaths from diarrhea were caused by inadequate WaSH practices in low- and middle-income countries (LMICs) in 2012.^18^ Despite the established role of safe WaSH in preventing diarrheal diseases,^19^ whether better WaSH practices can alleviate the potential association of floods with diarrhea risk is unknown. In this study, we used data from 43 LMICs from 2009 through 2019 to (1) investigate the association between floods and the risk of diarrhea in children younger than 5 years, (2) assess the possible compounding effect of drought before floods, and (3) explore potential effect modification by WaSH practices.

## Methods

This cross-sectional study was determined by the Yale University Institutional Review Board to be not human participant research and was exempt from ethical review. In this secondary data analysis, we used data from the Demographic and Health Surveys Program (DHS), which obtained verbal informed consent.^20^ We followed the Strengthening the Reporting of Observational Studies in Epidemiology (STROBE) reporting guideline.

### Diarrhea Data

We obtained individual-level information on diarrhea prevalence from the DHS, which has been conducting nationally representative household surveys in LMICs since 1984.^20^ Each woman of reproductive age (15-49 years) living in the surveyed household was asked about whether each of her children younger than 5 years had experienced diarrhea in the 2 weeks before the interview. We included 71 surveys conducted in 43 countries from 2009, when WaSH information from DHS surveys became fully available, through 2019. The DHS uses the geographic center of each survey cluster, randomly displaced by 2 to 10 km to protect anonymity, as the geocoded coordinate location for the selected households (approximately 25) within the cluster.^21^ Sociodemographic factors, including the child’s age and sex and mother’s education, were reported by the mother. We computed a wealth index that is comparable across surveys and countries based on household characteristics, as detailed previously.^10^ WaSH variables were reported by any knowledgeable person aged 15 years or older living in the household.

### Flood Data

We acquired flood data from the Dartmouth Flood Observatory,^22^ which has been documenting all large flood events globally since 1985 with their geographic information, start and end dates, and severity (large, very large, or extreme) according to the damage caused, affected area, and recurrence interval (eTable 1 in Supplement 1). We extracted all historical flood events occurring at the geographic location of each survey cluster during 2009 through 2019. We then matched the dates of each flood event with relevant dates for each child to determine flood exposure. We assumed, based on previous research, that floods could be associated with diarrhea risk for up to 8 weeks after each flood’s start date.^5^ However, given that mothers were queried about diarrhea occurrence during the 2 weeks before the interview, to ensure temporality (ie, that an exposed child experienced diarrhea after the flood start date), we excluded interview dates during the first 2 weeks after the start date of each flood; that is, we considered a child to be exposed to a flood if the interview occurred between 2 and 8 weeks after the start of the flood. Specifically, we defined 2 types of effect lag periods: single-week lags (ie, lag 3 weeks, lag 4 weeks, and so on to lag 8 weeks) and cumulative week lags (ie, lag 3 weeks, lag 3-4 weeks, through to lag 3-8 weeks). Thus, for example, we considered a child to be exposed to a flood during lag 3 weeks if the interview occurred during the third week (ie, between 15 and 21 days) after the start date of the flood, and we considered a child to be exposed during cumulative lag 3 to 5 weeks if the interview was conducted during the third through fifth week after the start of the flood (eFigure 1 in Supplement 1). A child who did not meet these criteria was considered to be unexposed to floods.

It is important to reiterate that the lag refers to the interview date, with the possibility that for a given child, diarrhea occurred during any of the 14 days prior to the interview. Thus, lag 3 weeks, for example, encompasses the first 3 weeks after the start of the flood, lag 4 weeks encompasses weeks 2 to 4 after the start of the flood, and cumulative lag 3 to 5 weeks encompasses the first 5 weeks since the start of the flood. Collection of temperature and rainfall data and assessment of drought are described in eMethods 1 in Supplement 1.

### Statistical Analysis

We used binomial generalized linear mixed-effects logistic regression models to examine the association between diarrhea risk and exposure to floods, with nested random intercepts for country and survey cluster (eMethods 2 in Supplement 1). The country-level random intercepts were included due to possible cross-country differences in the association.^23^ Specifically, we regressed a binary indicator of diarrhea prevalence on a binary indicator of flood exposure, adjusting for baseline characteristics that included the child’s age and sex, mother’s education, rural or urban area of residence, and wealth index (in quintiles). Total rainfall in the month of interview was also included in the models, since it has been found to be associated with both floods and diarrhea risk.^5^ Categorical month of interview and a natural cubic spline for calendar year of interview with 3 degrees of freedom were included to control for seasonality and long-term trends, respectively. Two secondary analyses were further conducted using multilevel variables for flood severity (large, very large, or extreme) and duration (≤2 weeks or >2 weeks).

To test the possible compounding effect of preceding drought at the geographic coordinates of a given survey cluster at the start of the flood, we modeled a 3-level categorical composite indicator representing the combined effect of drought and floods: (1) nonexposure to floods, (2) exposure to floods but nonexposure to prior drought, and (3) exposure to floods and exposure to prior drought. We examined 4 drought time scales (3, 6, 12, and 24 months).

To investigate potential effect modification by WaSH variables, in separate models we incorporated a main effect for each WaSH variable along with an interaction term between the WaSH variable and the binary flood exposure indicator. The WaSH variables we examined were source of drinking water (improved, unimproved), round-trip time to collect water (water on premises, <30 minutes, ≥30 minutes), type of toilet facility (improved, unimproved), place to wash hands (fixed, mobile), availability of water at handwashing site (no, yes), availability of soap or detergent at handwashing site (no, yes), and water treatment for drinking (no, yes). Effect modification analyses were performed for cumulative lags.

The statistical significance of the difference between estimates in the flood severity and duration analyses and the drought-flood compound event analysis was tested by calculating the 95% CI as follows:
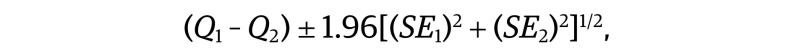
where *Q*_1_ and *Q*_2_ are the estimates for different categories of the variable of interest, and *SE*_1_ and *SE*_2_ are their respective SEs.^24^ The significance of heterogeneity across effect estimates in the WaSH effect modification analyses were tested by Cochran *Q* statistic in a meta-analysis.^25^

We performed several sensitivity analyses. First, we included in the models a natural cubic spline of monthly mean temperature in the month of interview with 3 degrees of freedom to evaluate the potential effect of temperature adjustment on the flood risk estimates. Second, we removed monthly total rainfall from the models. Third, we conducted multiple imputation (n = 5), implemented by multivariate imputation by chained equations,^26^ to impute each missing variable value in the main model. We then performed the regression analysis for each imputed data set and pooled their estimates.^10^ Fourth, we applied sampling weight in the models to account for the complex DHS survey design.^27^

All data analyses were performed between September 1 and December 31, 2022, using R, version 4.0.2 statistical software (R Foundation for Statistical Computing), with the MASS package for regression analysis. A 2-sided *P* < .05 was considered significant.

## Results

**Figure 1.**
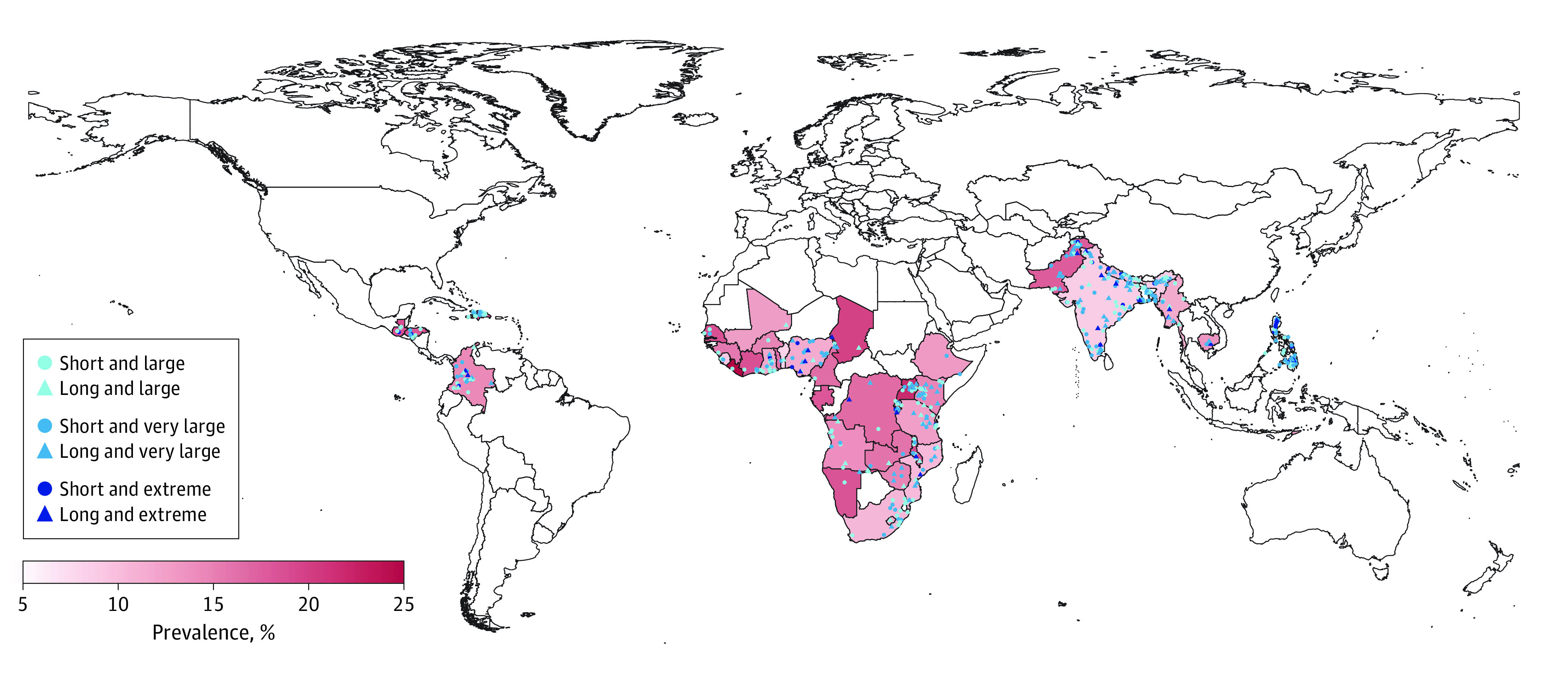
Prevalence of Diarrhea Among Children Younger Than 5 Years and the Centroid of Flood Events in 43 Low- and Middle-Income Countries During 2009-2019 The centroid of flood events is shown as the center point of a polygon that covers the flooded area. Short and long floods indicate those lasting 2 weeks or less and longer than 2 weeks, respectively.

A total of 914 097 children were included in 71 surveys. The overall prevalence of diarrhea among children younger than 5 years was 13.3% (120 304 of 904 197 children, with 9900 excluded due to missing diarrhea information) (Table 1), with 10 055 (1.1%) of these children exposed to floods during the first 8 weeks after the start of a flood. The highest diarrhea prevalence was observed in Liberia (24.6%), followed by Burundi (22.7%) and Uganda (21.4%) (Figure 1). Children were exposed to a total of 22 floods in 10 countries during the study period (eTable 2 in Supplement 1). Six of these floods were large, 12 were very large, and 4 were extreme. The duration of 10 floods was 2 weeks or less, and the duration of 12 floods was more than 2 weeks. More children were exposed to very large and extreme floods than to large floods and floods lasting for more than 2 weeks than to floods lasting 2 weeks or less (eTables 2 and 3 in Supplement 1). The number of children exposed to drought before floods increased with increasing drought time scale and with increasing cumulative lag period (eTable 4 in Supplement 1). The frequency of drought at different time scales during the study period is shown in eFigures 2 and 3 in Supplement 1.

**Table 1.** Participant Characteristics by Flood Exposure, Diarrhea Occurrence, and Water, Sanitation, and Hygiene Variables (N = 904 197)^a^

Characteristic	No. (%)^b^				
	Missing	Exposure at lag 3-8 wk		Diarrhea occurrence	
		Exposed	Unexposed	Yes	No
Total	0	10 055	894 142	120 304	783 893
Child’s sex	0				
Female		5026 (50.0)	438 536 (49.0)	57 176 (47.5)	386 386 (49.3)
Male		5029 (50.0)	455 606 (51.0)	63 128 (52.5)	397 507 (50.7)
Child’s age, mo	205 388 (22.7)				
<6		669 (9.9)	70 881 (10.2)	7536 (8.4)	64 014 (10.5)
6-23		2079 (30.9)	215 526 (31.1)	44 020 (49.3)	173 585 (28.5)
24-59		3989 (59.2)	405 665 (58.6)	37 678 (42.2)	371 976 (61.0)
Mother’s education	0				
No education		4407 (43.8)	317 159 (35.5)	43 494 (36.2)	278 072 (35.5)
Primary		2588 (25.7)	258 172 (28.9)	40 555 (33.7)	220 205 (28.1)
Secondary		2471 (24.6)	267 080 (29.9)	31 599 (26.3)	237 952 (30.4)
Higher		589 (5.9)	51 702 (5.8)	4650 (3.9)	47 641 (6.1)
Residence	0				
Urban		2677 (26.6)	259 262 (29.0)	33 483 (27.8)	228 456 (29.1)
Rural		7378 (73.4)	634 880 (71.0)	86 821 (72.2)	555 437 (70.9)
Wealth quintile	48 535 (5.4)				
First (lowest)		1893 (19.5)	169 985 (20.1)	27 145 (23.9)	144 733 (19.5)
Second		1940 (20.0)	168 635 (19.9)	24 974 (22.0)	145 601 (19.6)
Middle		2292 (23.6)	169 327 (20.0)	22 712 (20.0)	148 907 (20.1)
Fourth		1948 (20.1)	169 649 (20.1)	20 527 (18.1)	151 070 (20.4)
Fifth (highest)		1626 (16.8)	168 367 (19.9)	18 295 (16.1)	151 698 (20.4)
Source of drinking water	42 370 (4.7)				
Improved		2805 (28.7)	299 849 (35.2)	38 853 (33.9)	263 801 (35.3)
Unimproved		6981 (71.3)	552 192 (64.8)	75 911 (66.1)	483 262 (64.7)
Drinking water treatment	26 023 (2.9)				
No		7083 (70.5)	617 148 (71.1)	83 690 (72.0)	540 541 (70.9)
Yes		2960 (29.5)	250 983 (23.9)	32 610 (28.0)	221 333 (29.1)
Round-trip time to collect water	56 418 (6.2)				
On premises		3419 (35.1)	338 425 (40.4)	40 291 (35.5)	301 553 (41.1)
<30 min		4325 (44.4)	314 565 (37.5)	43 871 (38.7)	275 019 (37.5)
≥30 min		2006 (20.6)	185 039 (22.1)	29 304 (25.8)	157 741 (21.5)
Type of toilet facility	27 839 (3.1)				
Improved		2738 (38.8)	260 153 (43.0)	29 623 (36.2)	233 268 (44.0)
Unimproved		4316 (61.2)	344 396 (57.0)	52 295 (63.8)	296 417 (56.0)
Place to wash hands	99 967 (11.1)				
Fixed		4935 (60.4)	433 129 (64.0)	50 180 (58.0)	387 884 (64.8)
Mobile		3229 (39.6)	244 050 (36.0)	36 308 (42.0)	210 971 (35.2)
Water at handwashing site	360 734 (39.9)				
No		2466 (33.4)	148 469 (27.7)	22 147 (33.5)	128 788 (27.0)
Yes		4925 (66.6)	387 603 (72.3)	43 992 (66.5)	348 536 (73.0)
Soap or detergent at handwashing site	367 951 (40.7)				
No		4346 (64.3)	268 960 (50.8)	36 321 (55.2)	236 985 (50.4)
Yes		2414 (35.7)	260 526 (49.2)	29 478 (44.8)	233 462 (49.6)

^a^ Number of children younger than 5 years who had diarrhea in the 2 weeks before the study interview in 43 low- and middle-income countries during 2009-2019; 9900 children were excluded because of missing diarrhea information.

^b^ Denominators vary because of missing data in some categories. Some percentages may not add up to 100 because of rounding.

**Figure 2.**
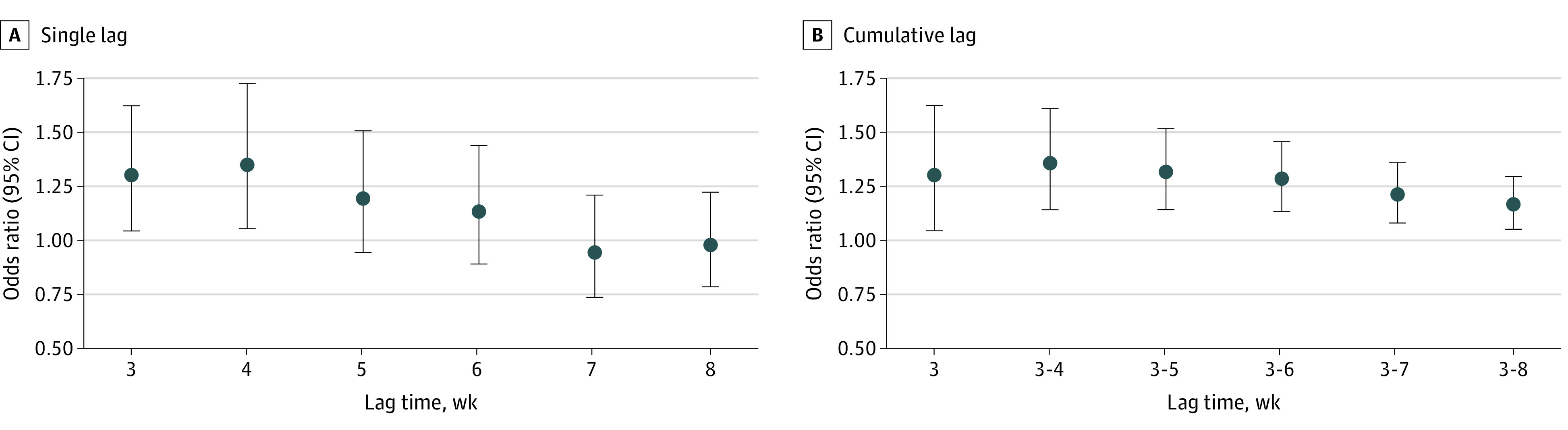
Associations Between Exposure to Floods and Risk of Diarrhea Among Children Younger Than 5 Years at Various Single and Cumulative Lag Times

**Figure 3.**
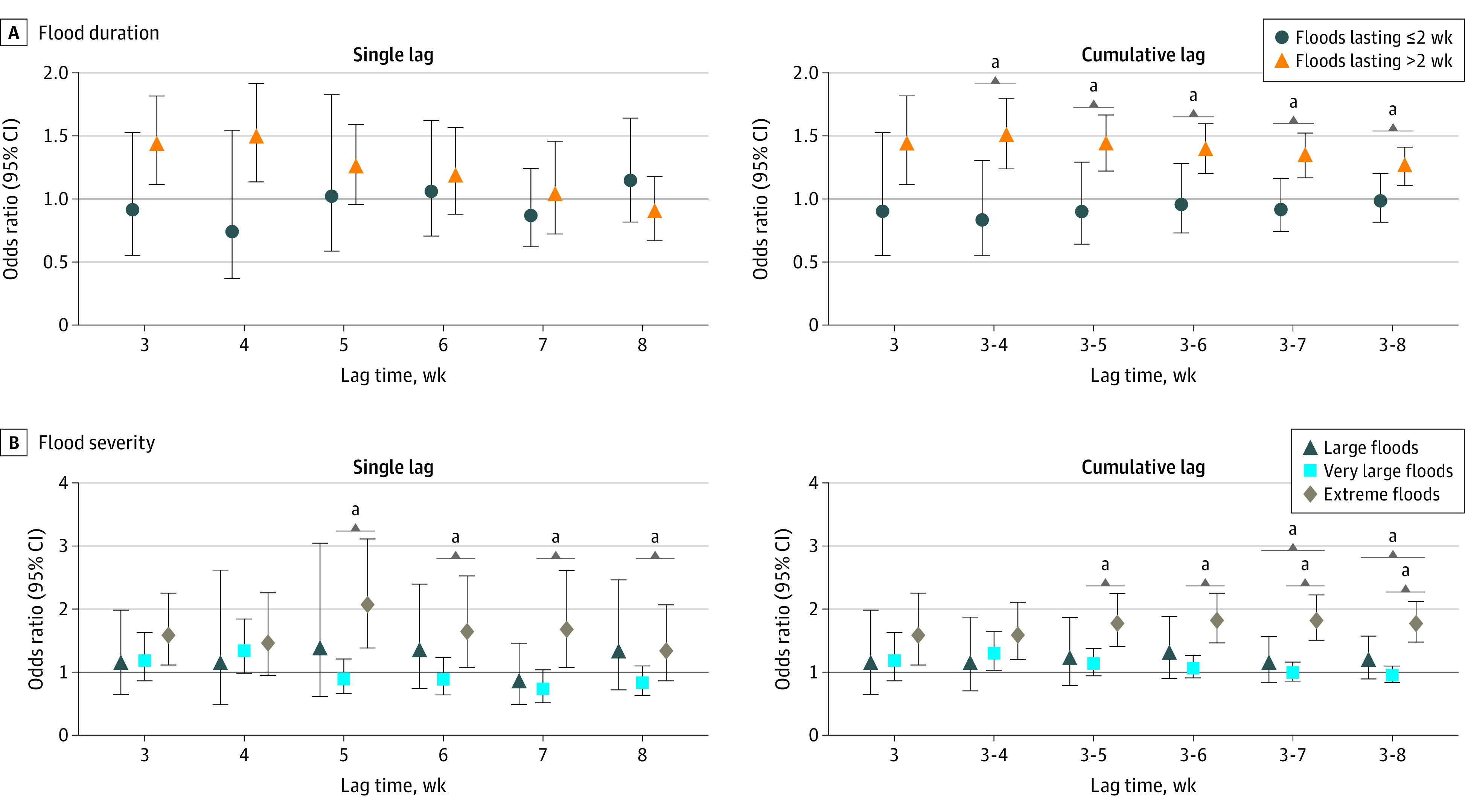
Associations Between Exposure to Floods and Risk of Diarrhea Among Children Younger Than 5 Years by Flood Duration and Severity Statistically significant differences for each lag period (*P* < .05) are based on the 95% CIs calculated as shown in the equation presented in the Statistical Analysis subsection of the Methods. ^a^Indicates statistically significant between-group differences.

Our main analyses were based on the complete data series (n = 639 250), which excluded children with missing values for diarrhea or baseline characteristics. In this series, 6365 children (mean [SD] age, 28.9 [17.2] months; 3214 boys [50.5%]; 3151 girls [49.5%]) were exposed to floods during the 8 weeks after a flood started, and the prevalence of diarrhea was 839 (13.2%) among children exposed to floods and 80 337 (12.7%) among those unexposed. We observed a significant positive association between exposure to floods and diarrhea prevalence at lag 3 weeks and lag 4 weeks (odds ratio [OR] at lag 4 weeks [ie, encompassing weeks 2-4 after the flood started], 1.35; 95% CI, 1.05-1.73) (Figure 2, single lag; eTable 5 in Supplement 1). The highest OR for cumulative lags was at cumulative lag 3 to 4 weeks (ie, encompassing the first 4 weeks after the flood started) (OR, 1.36; 95% CI, 1.14-1.61). The ORs for cumulative lags then gradually decreased but remained statistically significant through cumulative lag 3 to 8 weeks (Figure 2, cumulative lag; eTable 5 in Supplement 1). Significant associations were observed only for floods with a duration of more than 2 weeks (OR at lag 4 weeks, 1.47; 95% CI, 1.13-1.92) and for extreme floods (OR at lag 5 weeks, 2.07; 95% CI, 1.37-3.11), with significantly stronger associations than for less extreme or shorter-duration floods (which were not associated with diarrhea risk), particularly for cumulative and longer lags (Figure 3; eTable 5 in Supplement 1).

The OR for exposure to floods with prior drought was notably higher than that for floods with no prior drought, and the compounding effect of drought varied by drought time scale (Table 2; eFigure 4 in Supplement 1). Specifically, exposure to floods after a 3-month drought (OR, 2.21; 95% CI, 1.27-3.85) showed a significantly stronger association with diarrhea risk than flood exposure without prior drought (OR, 1.19; 95% CI, 0.93-1.51) at lag 3 weeks (*P* = .04 for difference in association) (eFigure 4 in Supplement 1) but not at longer cumulative lags. However, we found significantly stronger associations for floods after a 6-month drought compared with floods with no prior drought at cumulative lags up to cumulative lag 3 to 6 weeks (for difference in association: lag 3 weeks, *P* = .006; lag 3-4 weeks, *P* = .005; lag 3-5 weeks, *P* = .03; lag 3-6 weeks, *P* = .03) (eFigure 4 in Supplement 1). This compounding effect was observed for all cumulative lags up to cumulative lag 3 to 8 weeks, when there was a prior 12-month drought (OR, 1.42; 95% CI, 1.23-1.65) or 24-month drought (OR, 1.39; 95% CI, 1.21-1.60) (stronger association for floods with prior drought than floods without prior drought; *P* < .001 for difference in association for 2 drought time scales and for 6 lag periods) (eFigure 4 in Supplement 1). Notably, ORs across cumulative lags for floods with no prior 12- or 24-month drought were not significantly elevated (eFigure 4 in Supplement 1).

**Table 2.** Compounding Influence of Preceding Drought and Effect Modification by Water, Sanitation, and Hygiene Practices at Cumulative Lag 3 to 4 Weeks

	Odds ratio (95% CI)	*P* value^a^
Preceding drought		
3-mo Drought before floods		
No	1.29 (1.07-1.55)	.13
Yes	1.89 (1.20-2.99)	
6-mo Drought before floods		
No	1.16 (0.94-1.42)	.005
Yes	1.96 (1.45-2.66)	
12-mo Drought before floods		
No	1.00 (0.79-1.27)	<.001
Yes	1.96 (1.53-2.52)	
24-mo Drought before floods		
No	0.97 (0.76-1.25)	<.001
Yes	1.87 (1.48-2.37)	
Water, sanitation, and hygiene		
Water at handwashing site		
No	1.80 (1.34-2.41)	.03
Yes	1.17 (0.92-1.48)	
Round-trip time to collect water		
On premises	1.41 (1.09-1.83)	.03
<30 min	1.71 (1.36-2.16)	
≥30 min	0.93 (0.67-1.28)	
Source of drinking water		
Improved	1.47 (1.10-1.96)	.68
Unimproved	1.36 (1.12-1.66)	
Drinking water treatment		
No	1.33 (1.10-1.61)	.76
Yes	1.41 (1.06-1.86)	
Soap or detergent at handwashing site		
No	1.58 (1.24-2.01)	.15
Yes	1.18 (0.87-1.61)	
Place to wash hands		
Fixed	1.27 (1.01-1.60)	.25
Mobile	1.57 (1.19-2.08)	
Type of toilet facility		
Improved	1.33 (0.94-1.88)	.77
Unimproved	1.41 (1.17-1.70)	

^a^ *P* values denote the significance of heterogeneity across groups, as described in the Statistical Analysis subsection of the Methods.

We found a significantly higher OR among children living in households with no water available at the household’s handwashing site (OR at lag 3-4 weeks, 1.80; 95% CI, 1.34-2.41) than among children living in households with water availability (OR at lag 3-4 weeks, 1.17; 95% CI, 0.92-1.48) (for difference in association: lag 3 weeks, *P* = .01; lag 3-4 weeks, *P* = .01; lag 3-5 weeks, *P* = .04) (Table 2; eFigure 5 in Supplement 1). We also observed a significantly lower OR among children living in households with a 30-minute or greater round-trip time to collect water (OR at lag 3-4 weeks, 0.93; 95% CI, 0.67-1.28) than among children with less than a 30-minute round-trip time (OR at lag 3-4 weeks, 1.71; 95% CI, 1.36-2.16) (for difference in association: lag 3 weeks, *P* = .008; lag 3-4 weeks, *P* = .002; lag 3-5 weeks, *P* = .002; lag 3-6 weeks, *P* = .01; lag 3-7 weeks, *P* = .01; lag 3-8 weeks, *P* = .005) (Table 2; eFigure 6 in Supplement 1) or with water on premises (OR at lag 3-4 weeks, 1.41; 95% CI, 1.09-1.83; *P* = .04) (Table 2; eFigure 6 in Supplement 1). At lags 3 to 7 and 3 to 8 weeks, we found a significantly higher OR among children washing hands at a mobile site than among children washing hands at a fixed site (for difference in association: lag 3-7 weeks, *P* = .01; lag 3-8 weeks, *P* = .002) (eFigure 7 in Supplement 1). We found no significant effect modification for other WaSH variables (Table 2; eFigures 8-11 in Supplement 1).

The sensitivity analyses showed that our estimates of the association between flood exposure and diarrhea prevalence were mainly consistent and robust, although there was some attenuation of the association when monthly total precipitation was removed from the model and some enhancement of the association when DHS sampling weights were applied to the model (eFigures 12-15 in Supplement 1).

## Discussion

In this multicountry, cross-sectional study, we found a positive association between exposure to floods and risk of diarrhea among children younger than 5 years living in LMICs. The findings suggest that this association is driven by extreme flood events, floods that lasted more than 2 weeks, and floods preceded by droughts.

The major body of literature indicates a significant positive association between flood exposure and risk of diarrhea,^4^ with a few exceptions reporting null associations.^28,29^ However, a recent meta-analysis that pooled 14 studies found the overall association of floods with risk of diarrhea to be nonsignificant, despite a relatively strong effect estimate (risk ratio of 1.56).^5^ Our study found an association that was strongest for extreme or longer-duration floods, with less extreme or shorter-duration floods not associated with diarrhea risk.

Few studies have sufficiently addressed the delayed outcomes associated with floods, with reported lags depending on the temporal resolution of the analysis, including daily,^11^ weekly,^30^ monthly,^31^ and mixed scales.^32^ Generally, previous studies found a significant association during the first week after the end of floods.^11,30,32^ It is worth noting that these studies all used short-term time-series models. Our individual-level analysis uniquely defined cumulative lags that extended the exposure time window to 8 weeks after floods started. In addition, by using the single-lag windows, we estimated the highest OR during the second to fourth week after floods started. Differing from previous studies that reported a relatively short window by which the strongest association was observed, mostly within 1 week,^5,33,34^ our study findings suggest an extended influence of prolonged and larger floods. However, we note that due to the way the data on diarrhea occurrence were queried (ie, diarrhea during the 2 weeks before the interview), we were unable to examine associations at the daily level.

Floods are associated with diarrheal diseases via multiple pathways.^4^ Direct associations include pathogen transport and infrastructure failure, both of which cause contamination of drinking water sources. Excess rainfall and floods not only facilitate the proliferation of diarrheal pathogens in reservoirs^35^ but also mobilize and spread them from soil or environmental surfaces and subsurfaces to watersheds used for the drinking water supply.^4,36^ Potential backflows from the already-polluted water bodies to groundwater can lead to further contamination of drinking water sources.^37^ Sewage treatment facilities can be overloaded by flooding,^38^ and high turbidity levels in source waters can overwhelm the water supply system,^39^ which could result in an elevated risk of diarrheal diseases. Flood events could also be indirectly associated with diarrhea risk. By devastating crops and livestock, floods may substantially reduce agricultural yield and increase undernutrition,^4,40^ which could increase susceptibility to diarrheal infection, although the time course of this indirect association might be considerably longer than the aforementioned direct associations.

We found that prior drought conditions compounded the positive association of floods with diarrhea. In hydrology and atmospheric sciences, this phenomenon is referred to as drought-flood abrupt alternation, and it has been found to influence surface water quality.^41^ Some epidemiologic evidence has also shown that extreme precipitation following a relatively dry period may be associated with an increased risk of diarrhea.^5,13,14^ However, our study investigated for the first time the compounding influence of drought before floods by quantitatively assessing individual-level drought exposure using a comprehensive measure. Our findings also support the previously proposed concentration-runoff hypothesis^4,5,13,14^; that is, preceding drought conditions could facilitate the concentration of diarrheal pathogens in the environment, and the following flooding could flush the accumulated microorganisms into the floodwater, increasing the risk of personal exposure. The consistent compounding influence we observed of 12- or 24-month drought further supports the possible aggravating effect of longer-term persistent dry conditions.

### Limitations

Our study had several limitations. First, the diarrhea status of each child younger than 5 years was reported by the mother and, thus, is susceptible to inaccurate recall resulting in outcome misclassification. Second, although we used the strictest criteria in our exposure assessment to ensure the validity of the definition of an exposed child, the unknown exact disease-onset date could lead to exposure misclassification. Relatedly, we were only able to examine weekly, and not daily, lags. Third, we had data about whether a child was living in an area where a flood occurred but did not have direct evidence that a given child was exposed to a flood. Fourth, in the DHS’s sampling approach, if a cluster is inaccessible due to floods, it is deliberately excluded from the survey,^42^ potentially reducing the number of exposed children in our study and, therefore, reducing the study’s statistical power. Fifth, because survey cluster locations were randomly displaced by 2 to 10 km to protect privacy, flood exposure of children living close to the border of a flooded area might be misclassified, particularly for smaller floods.

## Conclusions

This cross-sectional study found an association between exposure to floods, particularly extreme and long-duration flooding, and risk of diarrhea among children younger than 5 years, especially when drought preceded floods. With the projected increasing frequency and intensity of floods and drought under climate change, greater collective efforts are warranted to build adaptative capacity to protect children’s health from floods in LMICs.

## Notes

Supplement 1. **eMethods 1.** Collection of Temperature and Rainfall Data and Assessment of Drought **eMethods 2.** Model Specification of Main Analysis **eTable 1.** Severity Class of Flood Events **eTable 2.** Flood Events That Children (Among All 639 250 Children in the Complete Data Series) Were Exposed to During 2009-2019 **eTable 3.** Numbers of Children (Among All 639 250 Children in the Complete Data Series) Exposed to Floods With Various Severity and Duration **eTable 4.** Number of Children (Among All 639 250 Children in the Complete Data Series) Exposed to 3-, 6-, 12-, and 24-Month Drought Before Exposure to Floods With Various Cumulative Lag Periods **eTable 5.** Odds Ratios and 95% CIs for Associations Between Exposure to Floods and Risk of Diarrhea Among Children Under 5, by Lag Period **eFigure 1.** Single Lag and Cumulative Lag Periods for the Interview Date in Relation to the Flood Start Date **eFigure 2.** Number of Months With 3-Month and 6-Month Drought in 43 Countries During 2009-2019 **eFigure 3.** Number of Months With 12-Month and 24-Month Drought in 43 Countries During 2009-2019 **eFigure 4.** Odds Ratios and 95% CIs for Associations Between Exposure to Floods and Risk of Diarrhea Among Children Under 5 by Preceding Drought and Cumulative Lag Period **eFigure 5.** Effect Modification of the Association Between Exposure to Floods and Risk of Diarrhea Among Children Under 5 by Water Availability at Handwashing Site, by Cumulative Lag Period (N = 406 759) **eFigure 6.** Effect Modification of the Association Between Exposure to Floods and Risk of Diarrhea Among Children Under 5 by Roundtrip Time to Collect Water, by Cumulative Lag Period (N = 607 179) **eFigure 7.** Effect Modification of the Association Between Exposure to Floods and Risk of Diarrhea Among Children Under 5 by Place to Wash Hands, by Cumulative Lag Period (N = 585 304) **eFigure 8.** Effect Modification of the Association Between Exposure to Floods and Risk of Diarrhea Among Children Under 5 by Source of Drinking Water, by Cumulative Lag Period (N = 618 875) **eFigure 9.** Effect Modification of the Association Between Exposure to Floods and Risk of Diarrhea Among Children Under 5 Water by Treatment Before Drinking, by Cumulative Lag Period (N = 621 020) **eFigure 10.** Effect Modification of the Association Between Exposure to Floods and Risk of Diarrhea Among Children Under 5 by Soap/Detergent Availability at Handwashing Site, by Cumulative Lag Period (N = 399 679) **eFigure 11.** Effect Modification of the Association Between Exposure to Floods and Risk of Diarrhea Among Children Under 5 by Type of Toilet Facilities, by Cumulative Lag Period (N = 618 875) **eFigure 12.** Association Between Exposure to Floods and Risk of Diarrhea Among Children Under 5 When Monthly Mean Temperature Was Included as an Adjustment Covariate in the Main Model (N = 639 250) **eFigure 13.** Association Between Exposure to Floods and Risk of Diarrhea Among Children Under 5 When Monthly Total Precipitation Was Removed From the Main Model (N = 639 250) **eFigure 14.** Association Between Exposure to Floods and Risk of Diarrhea Among Children Under 5 Using Multiple Imputation for Missing Values (N = 914 097) **eFigure 15.** Association Between Exposure to Floods and Risk of Diarrhea Among Children Under 5 Taking Into Account DHS Sampling Weights (N = 639 250)
eReferences

Supplement 2.
Data Sharing Statemen
