# Supplementary material for: Floods and Diarrhea Risk in Young Children in Low- and Middle-Income Countries
Source: JAMA Pediatr. 2023 Oct 2;177(11):1206–14. doi: 10.1001/jamapediatrics.2023.3964 (PMC10546297; doi:10.1001/jamapediatrics.2023.3964)
Supplement: Supplement 1. — eMethods 1. Collection of Temperature and Rainfall Data and Assessment of Drought eMethods 2. Model Specification of Main Analysis eTable 1. Severity Class of Flood Events eTable 2. Flood Events That Children (Among All 639 250 Children in the Complete Data Series) Were Exposed to During 2009-2019 eTable 3. Numbers of Children (Among All 639 250 Children in the Complete Data Series) Exposed to Floods With Various Severity and Duration eTable 4. Number of Children (Among All 639 250 Children in the Complete Data Series) Exposed to 3-, 6-, 12-, and 24-Month Drought Before Exposure to Floods With Various Cumulative Lag Periods eTable 5. Odds Ratios and 95% CIs for Associations Between Exposure to Floods and Risk of Diarrhea Among Children Under 5, by Lag Period eFigure 1. Single Lag and Cumulative Lag Periods for the Interview Date in Relation to the Flood Start Date eFigure 2. Number of Months With 3-Month and 6-Month Drought in 43 Countries During 2009-2019 eFigure 3. Number of Months With 12-Month and 24-Month Drought in 43 Countries During 2009-2019 eFigure 4. Odds Ratios and 95% CIs for Associations Between Exposure to Floods and Risk of Diarrhea Among Children Under 5 by Preceding Drought and Cumulative Lag Period eFigure 5. Effect Modification of the Association Between Exposure to Floods and Risk of Diarrhea Among Children Under 5 by Water Availability at Handwashing Site, by Cumulative Lag Period (N = 406 759) eFigure 6. Effect Modification of the Association Between Exposure to Floods and Risk of Diarrhea Among Children Under 5 by Roundtrip Time to Collect Water, by Cumulative Lag Period (N = 607 179) eFigure 7. Effect Modification of the Association Between Exposure to Floods and Risk of Diarrhea Among Children Under 5 by Place to Wash Hands, by Cumulative Lag Period (N = 585 304) eFigure 8. Effect Modification of the Association Between Exposure to Floods and Risk of Diarrhea Among Children Under 5 by Source of Drinking Water, by Cumulative Lag Period (N = [file jamapediatr-e233964-s001.pdf]

Supplemental Online Content

Wang P, Asare EO, Pitzer VE, Dubrow R, Chen K. Floods and diarrhea risk in young children in low- and middle-income countries. *JAMA Pediatr*. Published online October 2, 2023. doi:10.1001/jamapediatrics.2023.3964

eMethods 1. Collection of Temperature and Rainfall Data and Assessment of Drought

eMethods 2. Model Specification of Main Analysis

eTable 1. Severity Class of Flood Events

eTable 2. Flood Events That Children (Among All 639 250 Children in the Complete Data Series) Were Exposed to During 2009-2019

eTable 3. Numbers of Children (Among All 639 250 Children in the Complete Data Series) Exposed to Floods With Various Severity and Duration

eTable 4. Number of Children (Among All 639 250 Children in the Complete Data Series) Exposed to 3-, 6-, 12-, and 24-Month Drought Before Exposure to Floods With Various Cumulative Lag Periods

eTable 5. Odds Ratios and 95% CIs for Associations Between Exposure to Floods and Risk of Diarrhea Among Children Under 5, by Lag Period

eFigure 1. Single Lag and Cumulative Lag Periods for the Interview Date in Relation to the Flood Start Date

eFigure 2. Number of Months With 3-Month and 6-Month Drought in 43 Countries During 2009-2019

eFigure 3. Number of Months With 12-Month and 24-Month Drought in 43 Countries During 2009-2019

eFigure 4. Odds Ratios and 95% CIs for Associations Between Exposure to Floods and Risk of Diarrhea Among Children Under 5 by Preceding Drought and Cumulative Lag Period

eFigure 5. Effect Modification of the Association Between Exposure to Floods and Risk of Diarrhea Among Children Under 5 by Water Availability at Handwashing Site, by Cumulative Lag Period (N = 406 759)

eFigure 6. Effect Modification of the Association Between Exposure to Floods and Risk of Diarrhea Among Children Under 5 by Roundtrip Time to Collect Water, by Cumulative Lag Period (N = 607 179)

eFigure 7. Effect Modification of the Association Between Exposure to Floods and Risk of Diarrhea Among Children Under 5 by Place to Wash Hands, by Cumulative Lag Period (N = 585 304)

eFigure 8. Effect Modification of the Association Between Exposure to Floods and Risk of Diarrhea Among Children Under 5 by Source of Drinking Water, by Cumulative Lag Period (N = 618 875)

eFigure 9. Effect Modification of the Association Between Exposure to Floods and Risk of Diarrhea Among Children Under 5 by Water Treatment Before Drinking, by Cumulative Lag Period (N = 621 020)

eFigure 10. Effect Modification of the Association Between Exposure to Floods and Risk of Diarrhea Among Children Under 5 by Soap/Detergent Availability at Handwashing Site, by Cumulative Lag Period (N = 399 679)

eFigure 11. Effect Modification of the Association Between Exposure to Floods and Risk of Diarrhea Among Children Under 5 by Type of Toilet Facilities, by Cumulative Lag Period (N = 618 875)

eFigure 12. Association Between Exposure to Floods and Risk of Diarrhea Among Children Under 5 When Monthly Mean Temperature Was Included as an Adjustment Covariate in the Main Model (N = 639 250)

eFigure 13. Association Between Exposure to Floods and Risk of Diarrhea Among Children Under 5 When Monthly Total Precipitation Was Removed From the Main Model (N = 639 250)

eFigure 14. Association Between Exposure to Floods and Risk of Diarrhea Among Children Under 5 Using Multiple Imputation for Missing Values (N = 914 097)

eFigure 15. Association Between Exposure to Floods and Risk of Diarrhea Among Children Under 5 Taking Into Account DHS Sampling Weights (N = 639 250)

eReferences

This supplemental material has been provided by the authors to give readers additional information about their work.

© 2023 Wang P et al. *JAMA Pediatrics.*

eMethods 1. Collection of Temperature and Rainfall Data and Assessment of Drought

We downloaded and rescaled monthly mean temperature and total rainfall at a resolution of 0.1° (~10×10 km) during the same study period from the fifth generation European Centre for Medium-Range Weather Forecasts atmospheric reanalysis of the global climate (ERA5-Land).[1] We calculated our drought indicator following the methodology of our previous work.[2] Briefly, we assessed drought condition by the standardized precipitation evapotranspiration index (SPEI), which offers a more accurate and robust measure than precipitation alone by computing the climatic water balance between the available water content of soil and vegetation and the atmospheric evaporative demand.[3] We linked gridded 3-month, 6-month,12-month, and 24-month SPEI, monthly mean temperature, and monthly total rainfall with each child according to the geographic coordinates of each survey cluster and the month and year when the mother was interviewed. Exposure to drought was defined as SPEI ≤ -0.5 and non-exposure to drought was defined as SPEI > -0.5.[4]

© 2023 Wang P et al. *JAMA Pediatrics.*

eMethods 2. Model Specification of Main Analysis

We employed binomial generalized linear mixed effects logistic regression models to quantify the associations between exposure to floods and risk of diarrhea among children:

$$Ln(\frac{P_i}{1-P_i}) = \beta_0 + \beta_1 X_j + \beta_2 COV_i + \varphi_j + \lambda + \omega_j$$

where i is the index for each individual child and j is the DHS survey cluster in a specific country. P_i is the probability of having diarrhea. X_j is the binary indicator representing flood exposure (yes or no) for cluster j. COV_i is a matrix of individual and household baseline characteristics, including child's sex and age, maternal education, urban/rural residence, and wealth index. φ_j is the monthly total rainfall in the month of the interview at cluster j. λ is a matrix of categorical survey month and a natural cubic spline of survey year with three degrees of freedom to adjust for seasonality and long-term trend, respectively. ω_j denotes the nested random intercepts for survey cluster and country, accounting for cross-cluster and cross-country differences.

© 2023 Wang P et al. *JAMA Pediatrics.*

eTable 1. Severity Class of Flood Events

Severity	Definition
Large flood events	Causing substantial damage to structures or agriculture, loss of life, and/or having a reported interval of 1–2 decades since the occurrence of a similar event.
Very large flood events	Having an estimated recurrence interval of more than 2 decades but less than 100 years, and/or having a local recurrence interval of 1–2 decades and impacting a vast geographic region exceeding 5000 square kilometers.
Extreme flood events	Characterized by an estimated recurrence interval surpassing 100 years.

Note: This classification method has been consistently adopted by the Dartmouth Flood Observatory.[5]

eTable 2. Flood Events That Children (Among All 639 250 Children in the Complete Data Series) Were Exposed to During 2009-2019

	Start date	Country affected	Duration (days)	Severity	Number of children exposed during lag 3–8 weeks
1	2009/11/10	Tanzania	3	Large	103
2	2009/12/25	Tanzania	2	Large	214
3	2010/08/01	Burkina Faso	40	Extreme	219
4	2011/08/20	Uganda	19	Very large	18
5	2011/11/09	Uganda	41	Very large	3
6	2012/04/08	Haiti	18	Very large	908
7	2012/08/24	Senegal	5	Very large	257
8	2014/07/13	Bangladesh	1	Very large	9
9	2014/08/20	Bangladesh	19	Very large	626
10	2015/05/17	India	4	Large	118
11	2015/06/02	India	27	Very large	298
12	2015/07/15	India	35	Extreme	214
13	2016/01/14	Tanzania	15	Large	187
14	2016/04/04	Ethiopia	9	Very large	601
15	2016/04/20	India	11	Very large	142
16	2016/06/29	India	58	Very large	657
17	2016/07/07	India	27	Very large	35
18	2016/07/15	India	69	Very large	302
19	2016/07/25	South Africa	9	Large	4
20	2018/07/13	Nigeria	4	Extreme	202
21	2018/08/20	Nigeria	43	Extreme	1016
22	2019/09/10	Senegal	10	Large	232

© 2023 Wang P et al. *JAMA Pediatrics.*

eTable 3. Numbers of Children (Among All 639 250 Children in the Complete Data Series) Exposed to Floods With Various Severity and Duration

Lag period	All floods (% of all children)	Large floods	Very large floods	Extreme floods	Floods ≤2 weeks	Floods >2 weeks
Single lag						
3 weeks	1190 (0.19)	209	565	416	216	974
4 weeks	839 (0.13)	69	510	260	123	716
5 weeks	1008 (0.16)	82	681	245	176	832
6 weeks	1048 (0.16)	143	655	250	311	737
7 weeks	1047 (0.16)	210	612	225	532	515
8 weeks	1233 (0.19)	145	833	255	524	709
Cumulative lag						
3 weeks	1190 (0.19)	209	565	416	216	974
3–4 weeks	2029 (0.32)	278	1075	676	339	1690
3–5 weeks	3037 (0.48)	360	1756	921	515	2522
3–6 weeks	4085 (0.64)	503	2411	1171	826	3259
3–7 weeks	5132 (0.80)	713	3023	1396	1358	3774
3–8 weeks	6365 (1.00)	858	3856	1651	1882	4483

© 2023 Wang P et al. *JAMA Pediatrics.*

eTable 4. Number of Children (Among All 639 250 Children in the Complete Data Series) Exposed to 3-, 6-, 12-, and 24-Month Drought Before Exposure to floods During Various Cumulative Lag Periods

Lag period	Timescale of drought	Unexposed to drought (diarrhea/non-diarrhea)	Exposed to drought (diarrhea/non-diarrhea)
3 weeks	3-month	1061 (135/926)	129 (26/103)
	6-month	811 (103/708)	379 (58/321)
	12-month	707 (80/627)	483 (81/402)
	24-month	609 (68/541)	581 (93/488)
3–4 weeks	3-month	1789 (254/1535)	240 (38/202)
	6-month	1362 (198/1164)	667 (94/573)
	12-month	1102 (153/949)	927 (139/788)
	24-month	980 (137/843)	1049 (155/894)
3–5 weeks	3-month	2594 (370/2224)	443 (69/374)
	6-month	1995 (308/1687)	1042 (131/911)
	12-month	1661 (248/1413)	1376 (191/1185)
	24-month	1473 (220/1253)	1564 (219/1345)
3–6 weeks	3-month	3310 (454/2856)	775 (108/667)
	6-month	2573 (383/2190)	1512 (179/1333)
	12-month	2091 (293/1798)	1994 (269/1725)
	24-month	1819 (252/1567)	2266 (310/1956)
3–7 weeks	3-month	3880 (526/3354)	1252 (144/1108)
	6-month	3167 (458/2709)	1965 (212/1753)
	12-month	2554 (341/2213)	2578 (329/2249)
	24-month	2223 (299/1924)	2909 (371/2538)
3–8 weeks	3-month	4787 (669/4118)	1578 (170/1408)
	6-month	4077 (606/3471)	2288 (233/2055)
	12-month	3203 (446/2757)	3162 (393/2769)
	24-month	2862 (407/2455)	3503 (432/3071)

© 2023 Wang P et al. *JAMA Pediatrics.*

eTable 5. Odds Ratios and 95% CIs for Associations Between Exposure to Floods and Risk of Diarrhea Among Children Under 5, by Lag Period

Lag period	All floods	Floods ≤2 weeks	Floods >2 weeks	Large floods	Very large floods	Extreme floods
Single lag						
3 weeks	**1.30 (1.04, 1.62)**	0.91 (0.55, 1.52)	**1.42 (1.11, 1.81)**	1.12 (0.63, 1.98)	1.17 (0.85, 1.62)	**1.58 (1.11, 2.24)**
4 weeks	**1.35 (1.05, 1.73)**	0.75 (0.36, 1.54)	**1.47 (1.13, 1.92)**	1.11 (0.47, 2.62)	1.33 (0.97, 1.83)	1.45 (0.94, 2.25)
5 weeks	1.19 (0.95, 1.51)	1.03 (0.58, 1.83)	1.23 (0.95, 1.59)	1.36 (0.61, 3.04)	0.88 (0.65, 1.20)	**2.07 (1.37, 3.11)**
6 weeks	1.13 (0.89, 1.44)	1.06 (0.70, 1.62)	1.17 (0.87, 1.56)	1.32 (0.73, 2.39)	0.88 (0.63, 1.23)	**1.64 (1.06, 2.52)**
7 weeks	0.94 (0.74, 1.21)	0.88 (0.62, 1.24)	1.02 (0.72, 1.45)	0.83 (0.48, 1.45)	0.72 (0.50, 1.03)	**1.67 (1.07, 2.61)**
8 weeks	0.98 (0.79, 1.22)	1.15 (0.81, 1.64)	0.88 (0.66, 1.18)	1.32 (0.71, 2.46)	0.82 (0.62, 1.09)	1.33 (0.86, 2.06)
Cumulative lag						
3 weeks	**1.30 (1.04, 1.62)**	0.91 (0.55, 1.52)	**1.42 (1.11, 1.81)**	1.12 (0.63, 1.98)	1.17 (0.85, 1.62)	**1.58 (1.11, 2.24)**
3–4 weeks	**1.36 (1.14, 1.61)**	0.84 (0.55, 1.30)	**1.49 (1.24, 1.80)**	1.13 (0.69, 1.85)	**1.28 (1.01, 1.62)**	**1.57 (1.18, 2.09)**
3–5 weeks	**1.32 (1.14, 1.52)**	0.91 (0.64, 1.29)	**1.42 (1.22, 1.66)**	1.20 (0.78, 1.85)	1.12 (0.92, 1.36)	**1.76 (1.38, 2.23)**
3–6 weeks	**1.28 (1.13, 1.46)**	0.97 (0.73, 1.28)	**1.38 (1.20, 1.59)**	1.28 (0.88, 1.87)	1.05 (0.89, 1.25)	**1.80 (1.45, 2.23)**
3–7 weeks	**1.21 (1.08, 1.36)**	0.93 (0.74, 1.16)	**1.33 (1.17, 1.52)**	1.12 (0.82, 1.54)	0.98 (0.84, 1.14)	**1.81 (1.48, 2.21)**
3–8 weeks	**1.17 (1.05, 1.29)**	0.99 (0.81, 1.20)	**1.25 (1.10, 1.41)**	1.17 (0.88, 1.55)	0.94 (0.81, 1.08)	**1.75 (1.45, 2.10)**

Note: bold font represents statistically significant result.

© 2023 Wang P et al. *JAMA Pediatrics.*

eFigure 1. Single Lag and Cumulative Lag Periods for the Interview Date in Relation to the Flood Start Date. On the interview date, mothers reported diarrhea occurrence during the two weeks before the interview.

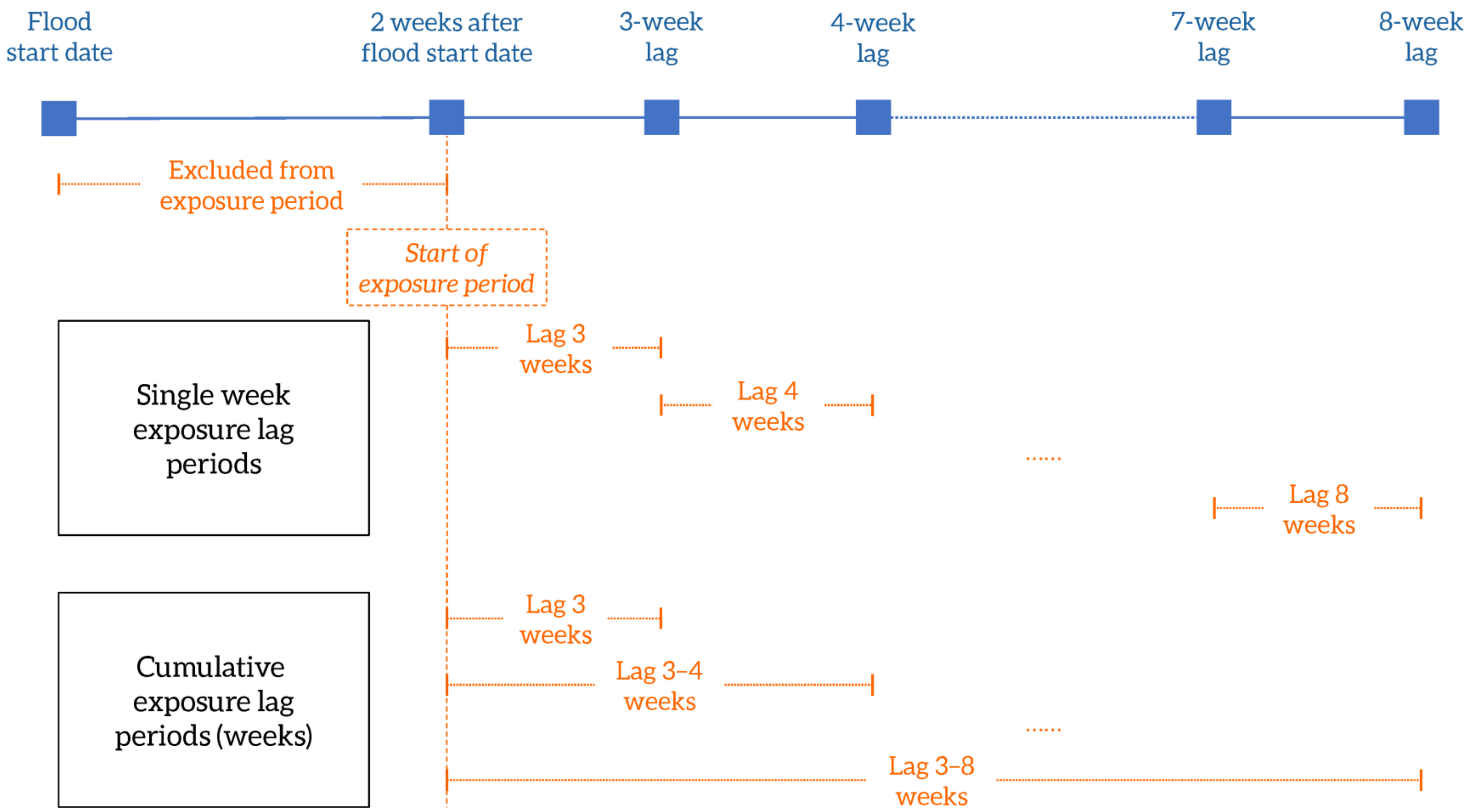

© 2023 Wang P et al. *JAMA Pediatrics.*

eFigure 2. Number of Months With 3-Month and 6-Month Drought in 43 Countries During 2009-2019

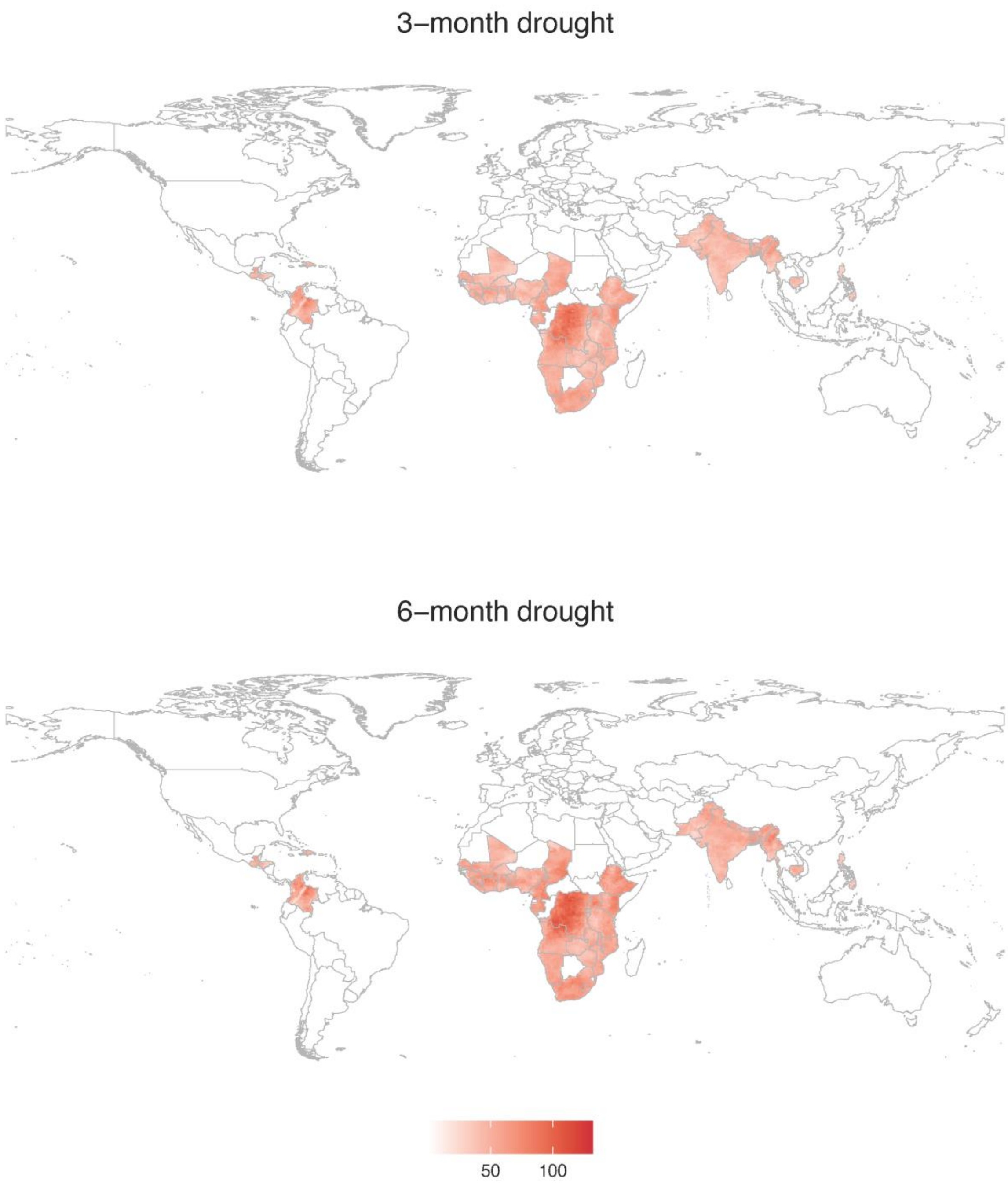

© 2023 Wang P et al. *JAMA Pediatrics.*

eFigure 3. Number of Months With 12-Month and 24-Month Drought in 43 Countries During 2009-2019

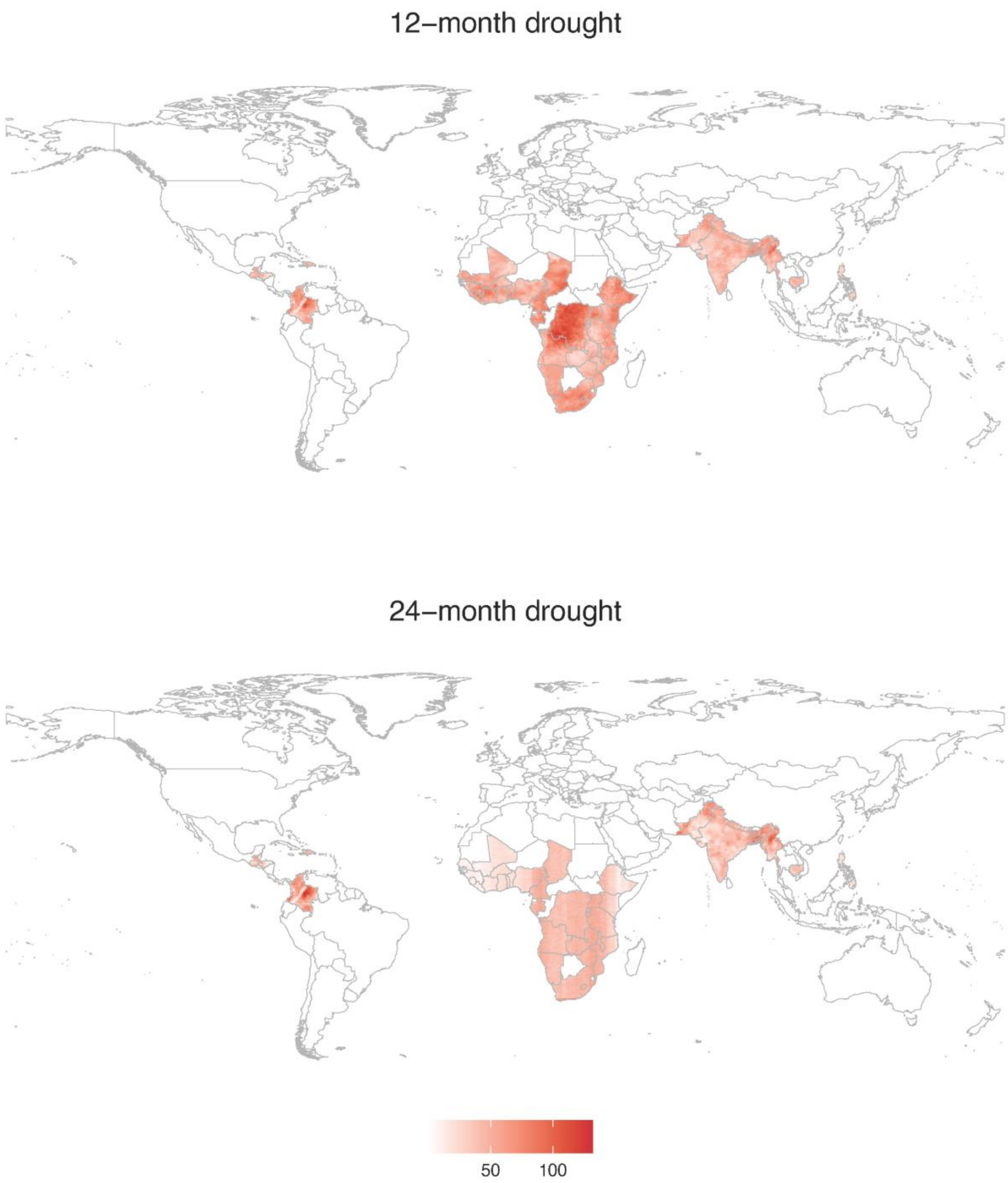

© 2023 Wang P et al. *JAMA Pediatrics.*

eFigure 4. Odds Ratios and 95% CIs for Associations Between Exposure to Floods and Risk of Diarrhea Among Children Under Age 5 by Preceding Drought and Cumulative Lag Period. Statistically significant differences [$p<0.05$] in the association are marked with an asterisk).

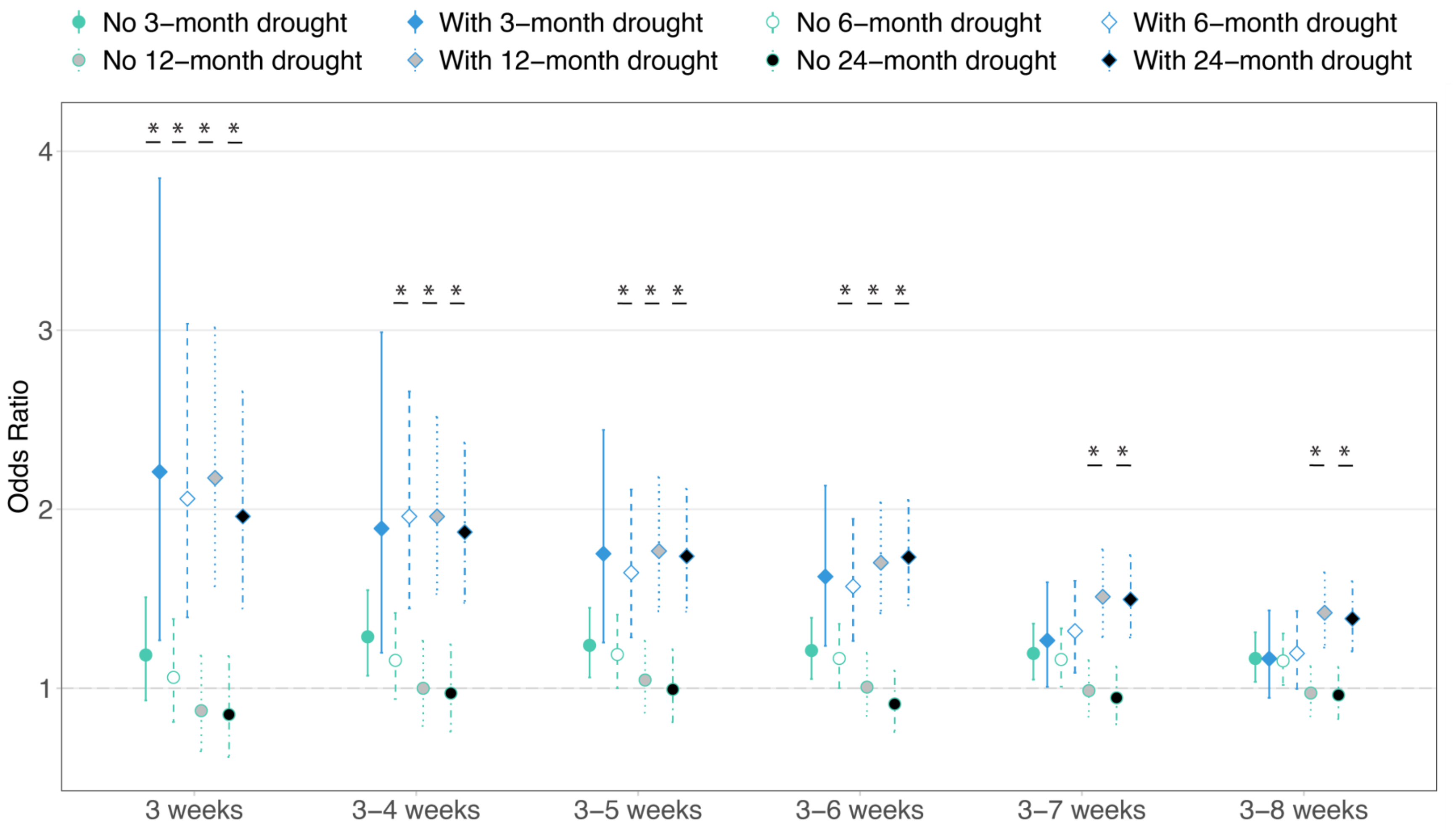

© 2023 Wang P et al. *JAMA Pediatrics.*

eFigure 5. Effect Modification of the Association Between Exposure to Floods and Risk of Diarrhea Among Children Under 5 by Water Availability at Handwashing Site, by Cumulative Lag Period (N = 406 759). Statistically significant differences [*p*<0.05] in the association are marked with an asterisk).

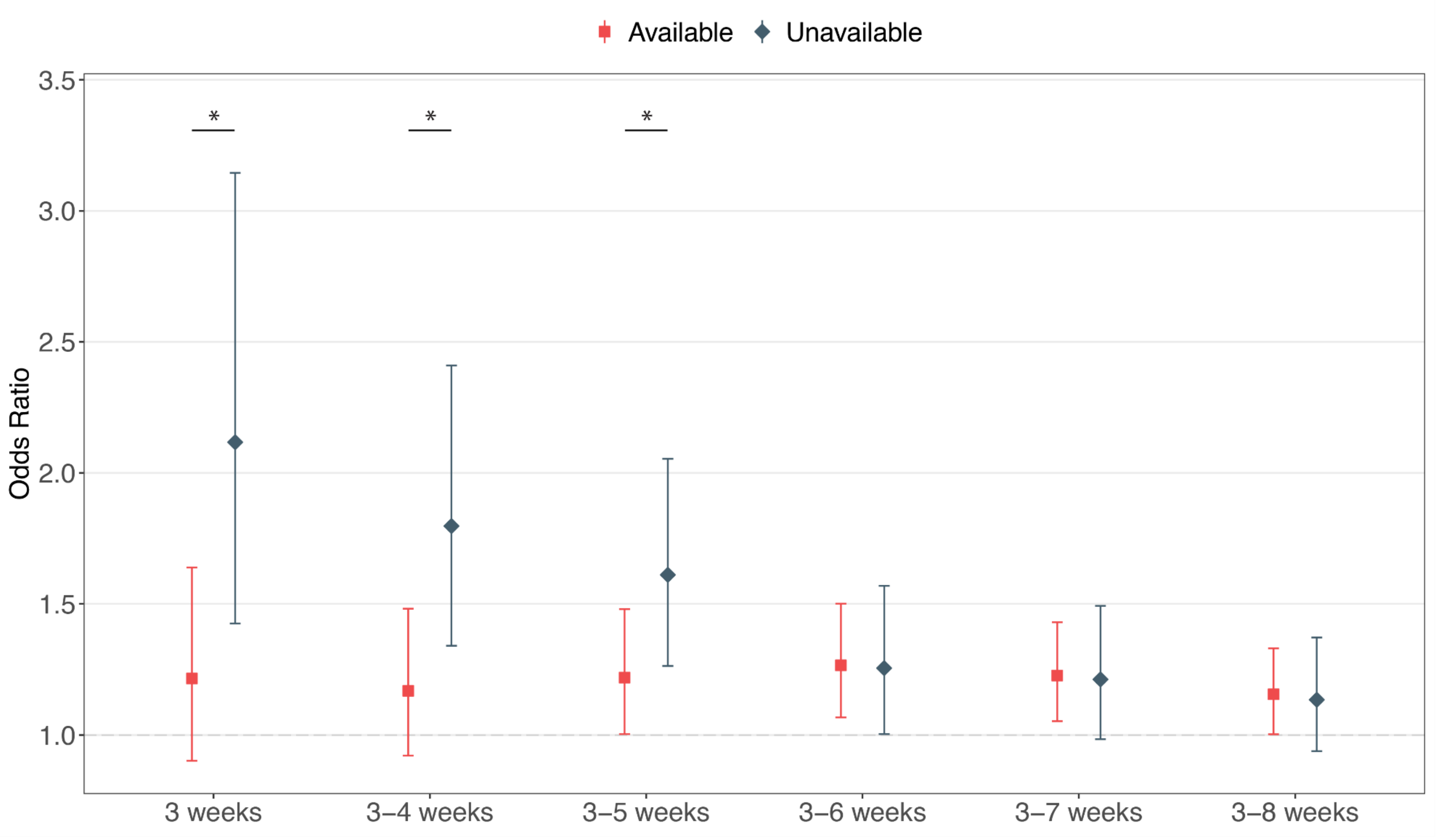

© 2023 Wang P et al. *JAMA Pediatrics.*

eFigure 6. Effect Modification of the Association Between Exposure to Floods and Risk of Diarrhea Among Children Under 5 by Roundtrip Time to Collect Water, by Cumulative Lag Period (N = 607 179). Statistically significant differences [*p*<0.05] in the association are marked with an asterisk).

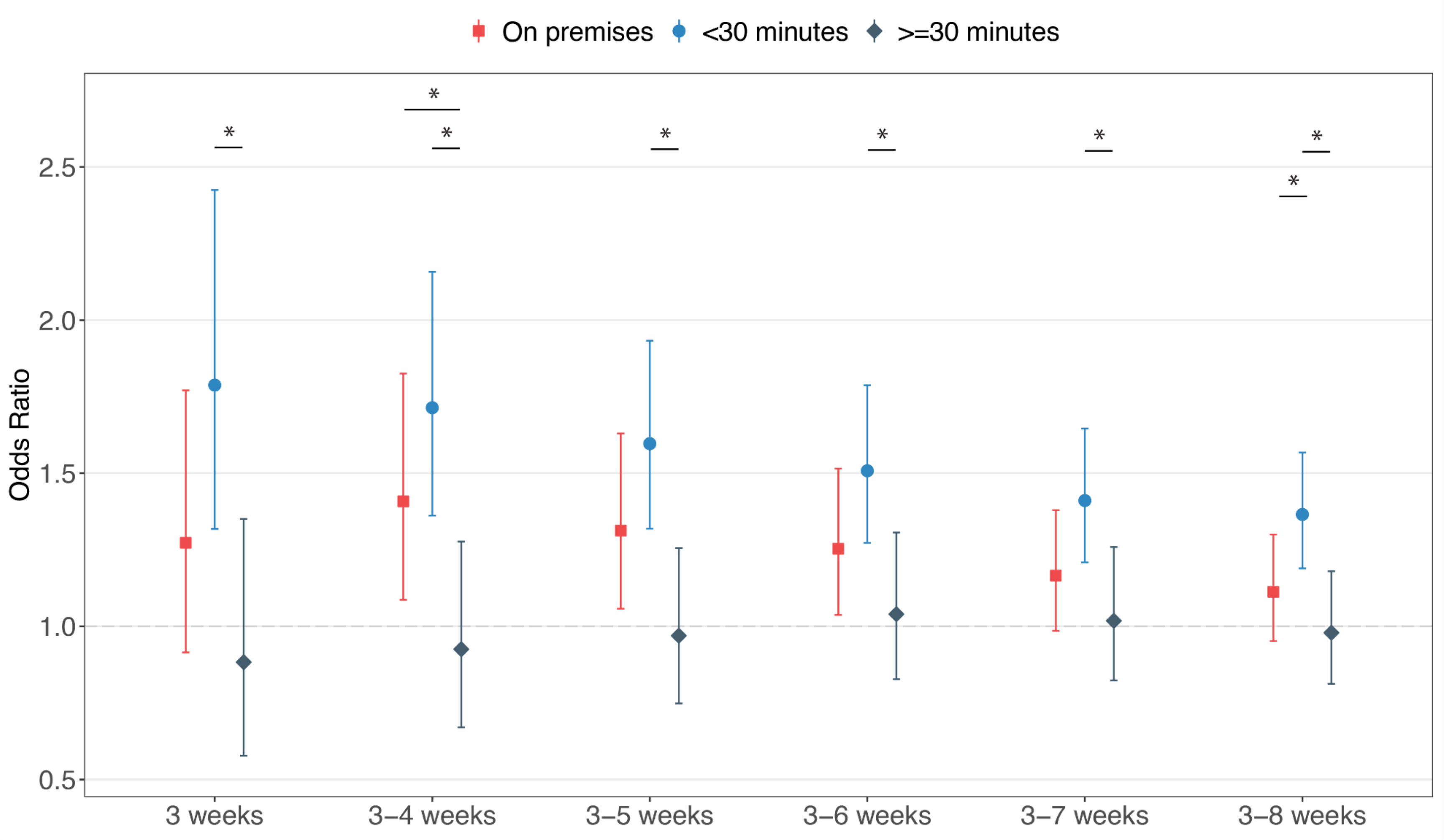

© 2023 Wang P et al. *JAMA Pediatrics.*

eFigure 7. Effect Modification of the Association Between Exposure to Floods and Risk of Diarrhea Among Children Under 5 by Place to Wash Hands, by Cumulative Lag Period (N = 585 304). Statistically significant differences [$p<0.05$] in the association are marked with an asterisk).

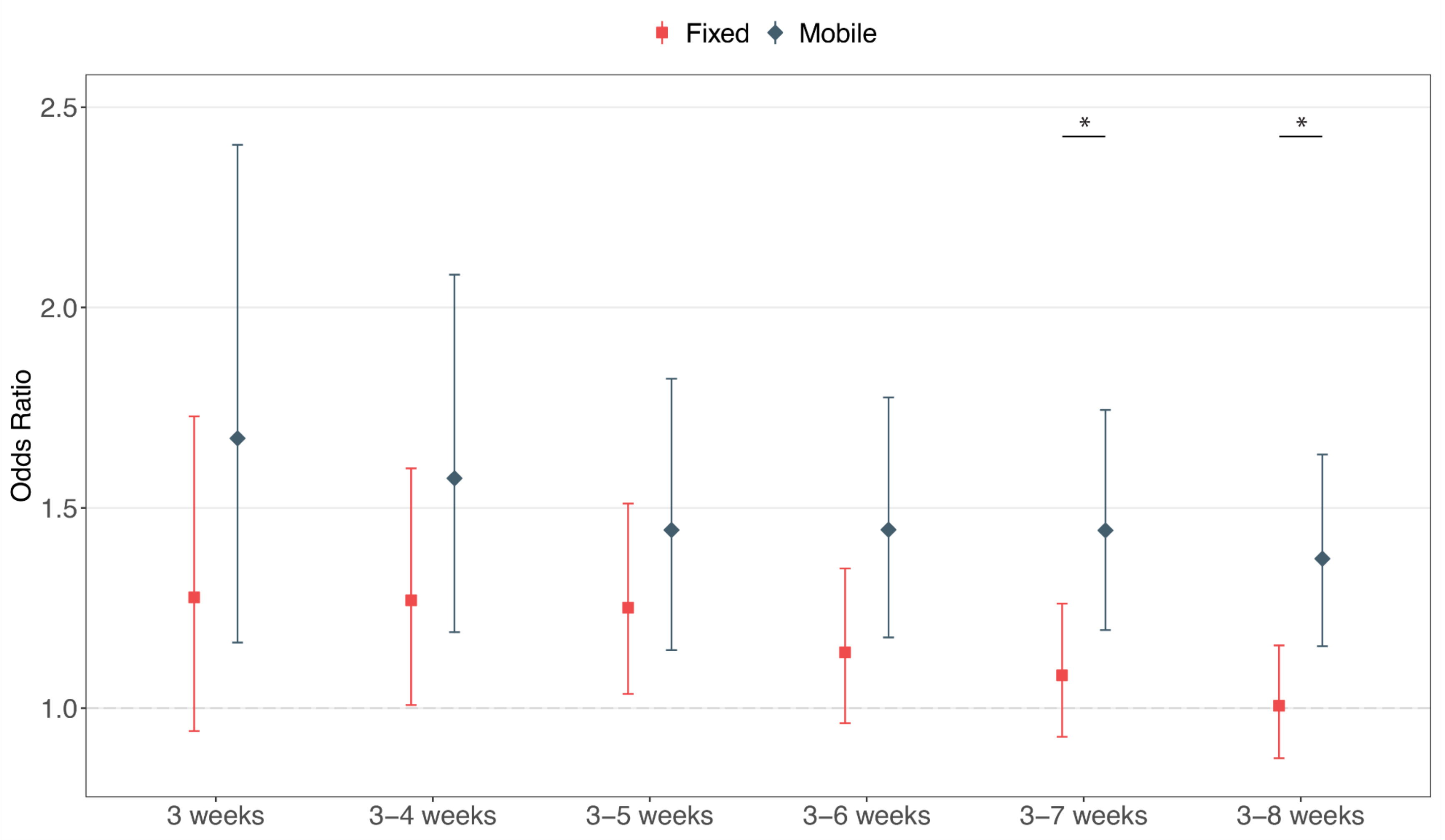

© 2023 Wang P et al. *JAMA Pediatrics.*

eFigure 8. Effect Modification of the Association Between Exposure to Floods and Risk of Diarrhea Among Children Under 5 by Source of Drinking Water, by Cumulative Lag Period (N = 618 875)

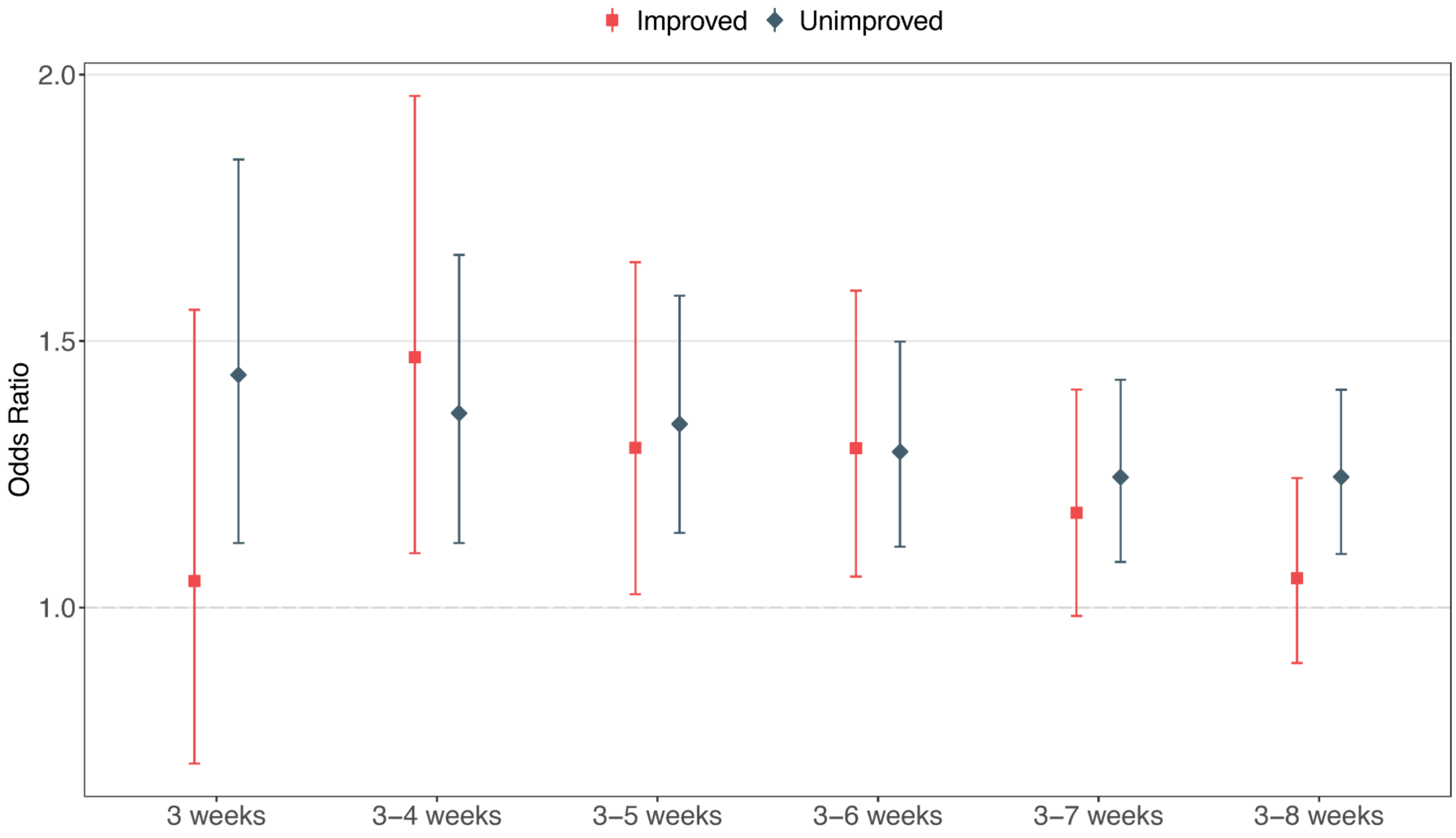

© 2023 Wang P et al. *JAMA Pediatrics.*

eFigure 9. Effect Modification of the Association Between Exposure to Floods and Risk of Diarrhea Among Children Under 5 by Water Treatment Before Drinking, by Cumulative Lag Period (N = 621 020)

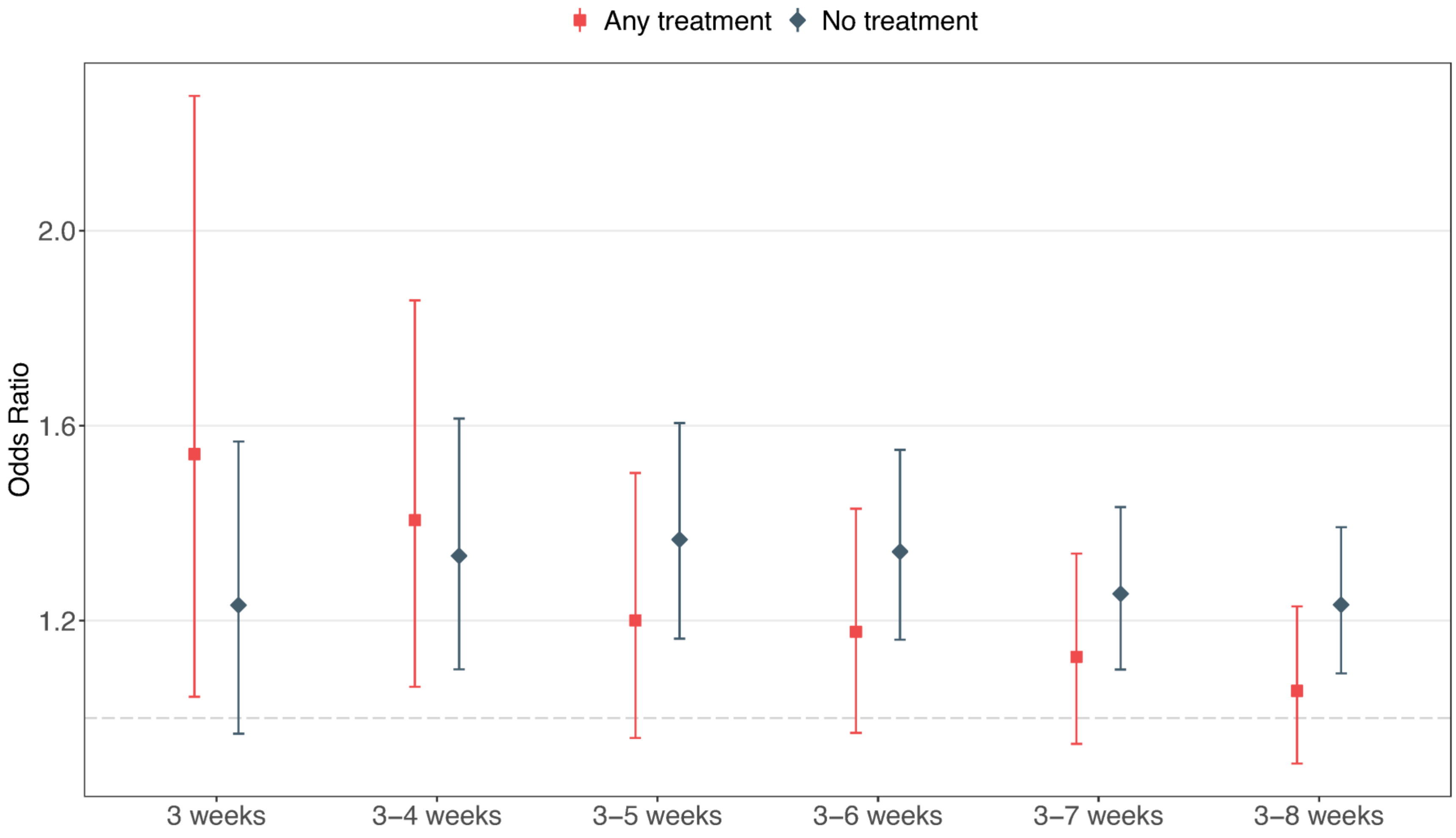

© 2023 Wang P et al. *JAMA Pediatrics.*

eFigure 10. Effect Modification of the Association Between Exposure to Floods and Risk of Diarrhea Among Children Under 5 by Soap/Detergent Availability at Handwashing Site, by Cumulative Lag Period (N = 399 679)

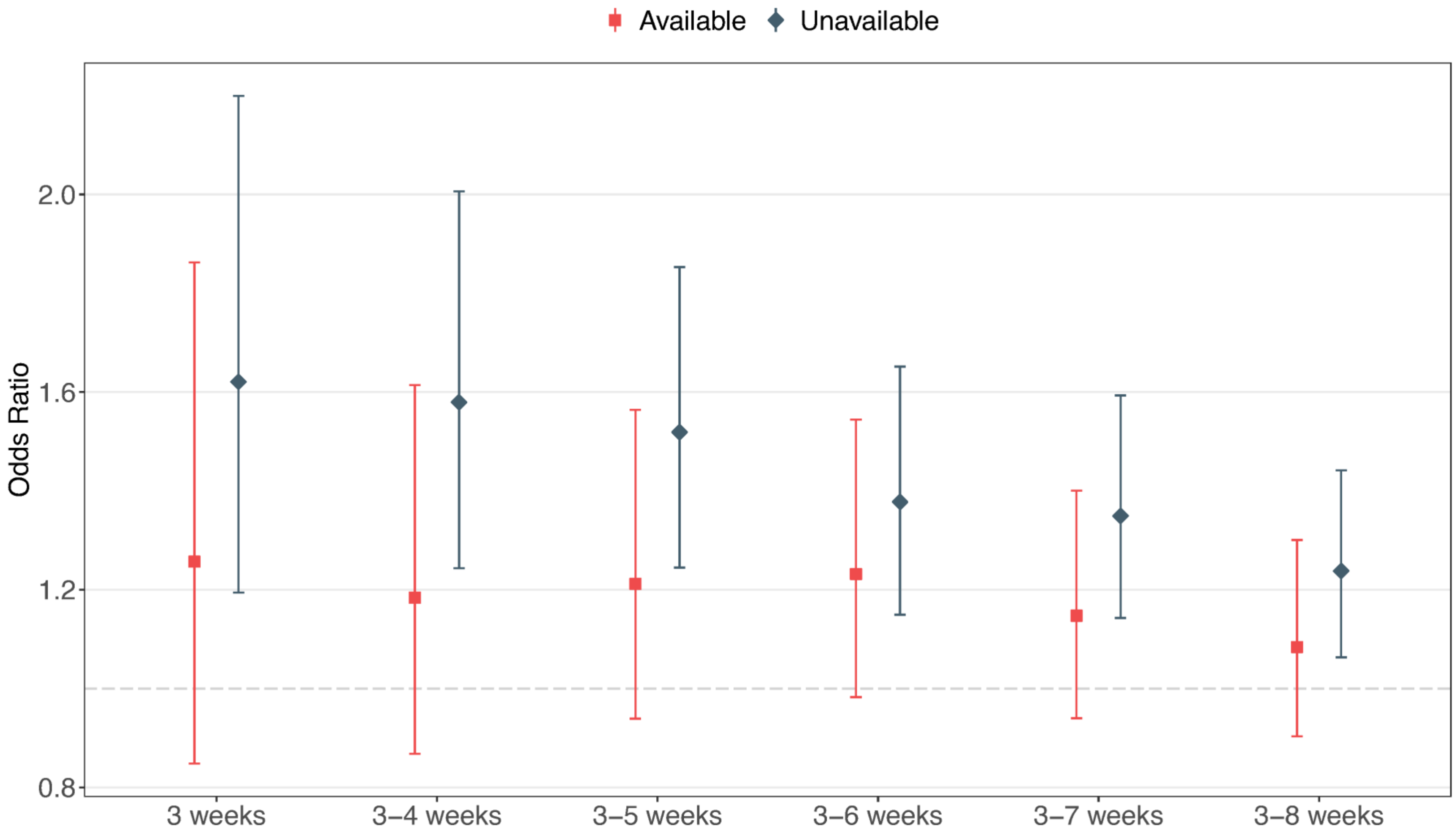

© 2023 Wang P et al. *JAMA Pediatrics.*

eFigure 11. Effect Modification of the Association Between Exposure to Floods and Risk of Diarrhea Among Children Under 5 by Type of Toilet Facilities, by Cumulative Lag Period (N = 618 875)

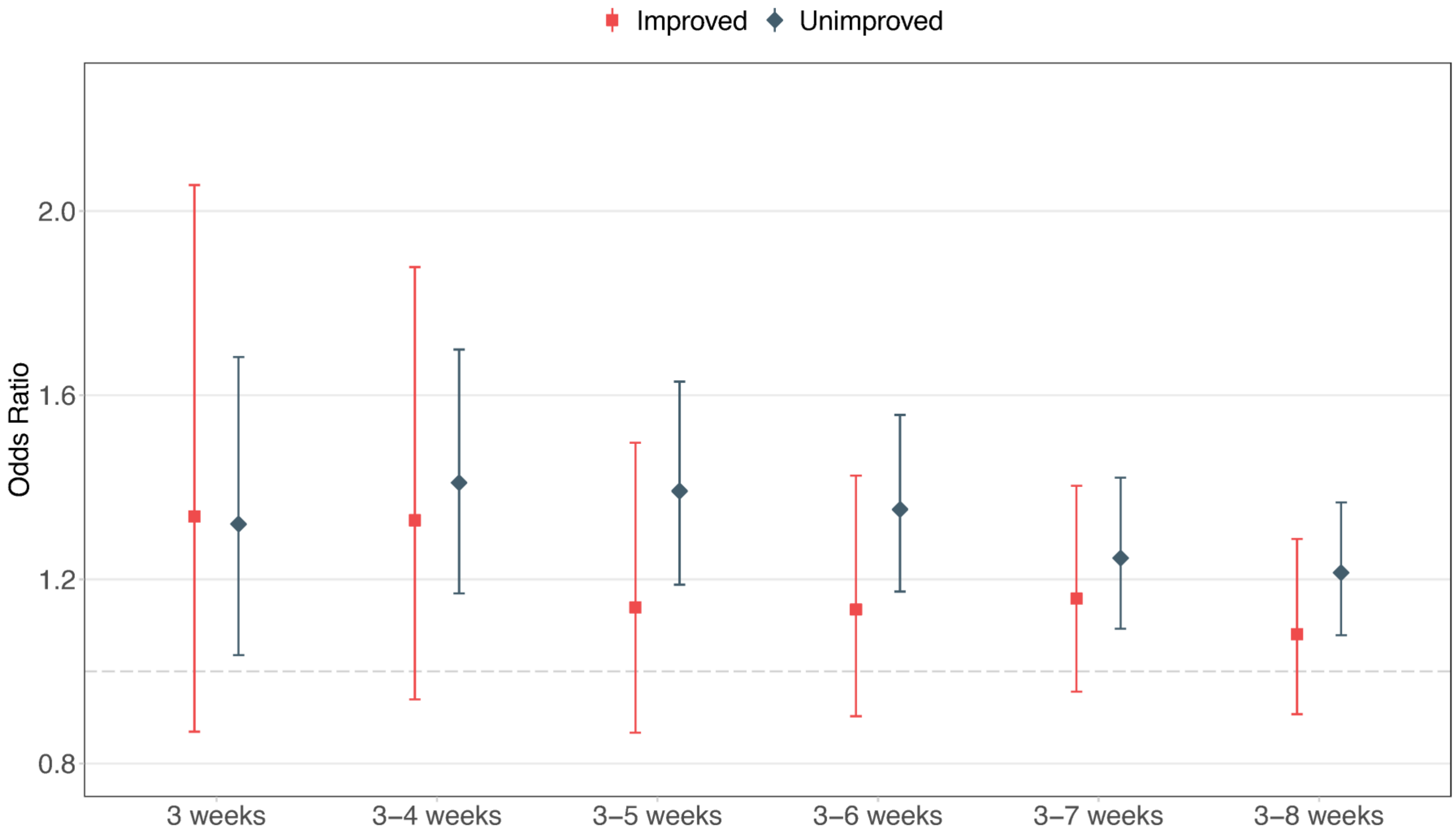

© 2023 Wang P et al. *JAMA Pediatrics.*

eFigure 12. Association Between Exposure to Floods and Risk of Diarrhea Among Children Under 5 When Monthly Mean Temperature Was Included as an Adjustment Covariate in the Main Model (N = 639 250)

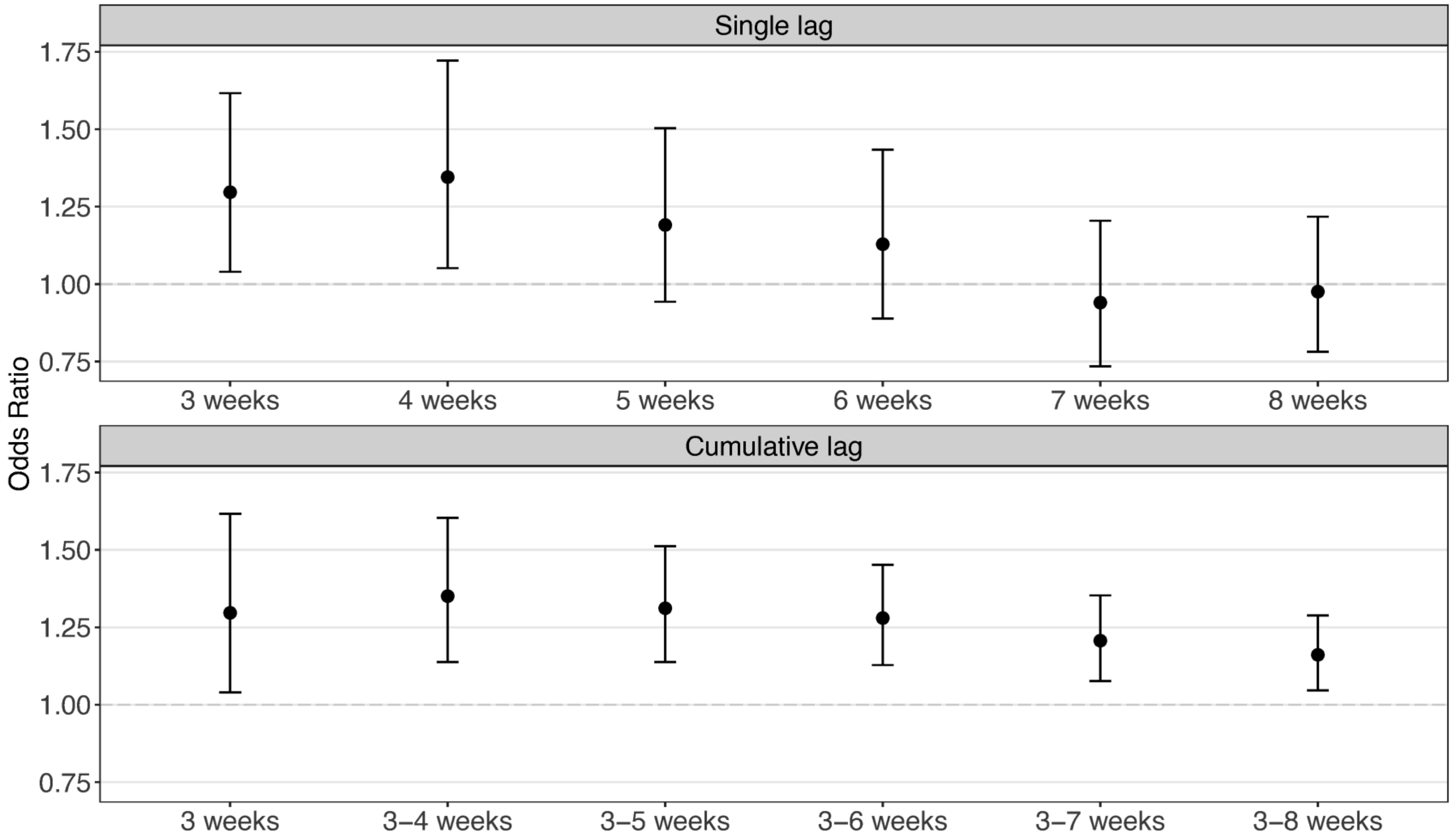

© 2023 Wang P et al. *JAMA Pediatrics.*

eFigure 13. Association Between Exposure to Floods and Risk of Diarrhea Among Children Under 5 When Monthly Total Precipitation Was Removed From the Main Model (N = 639 250)

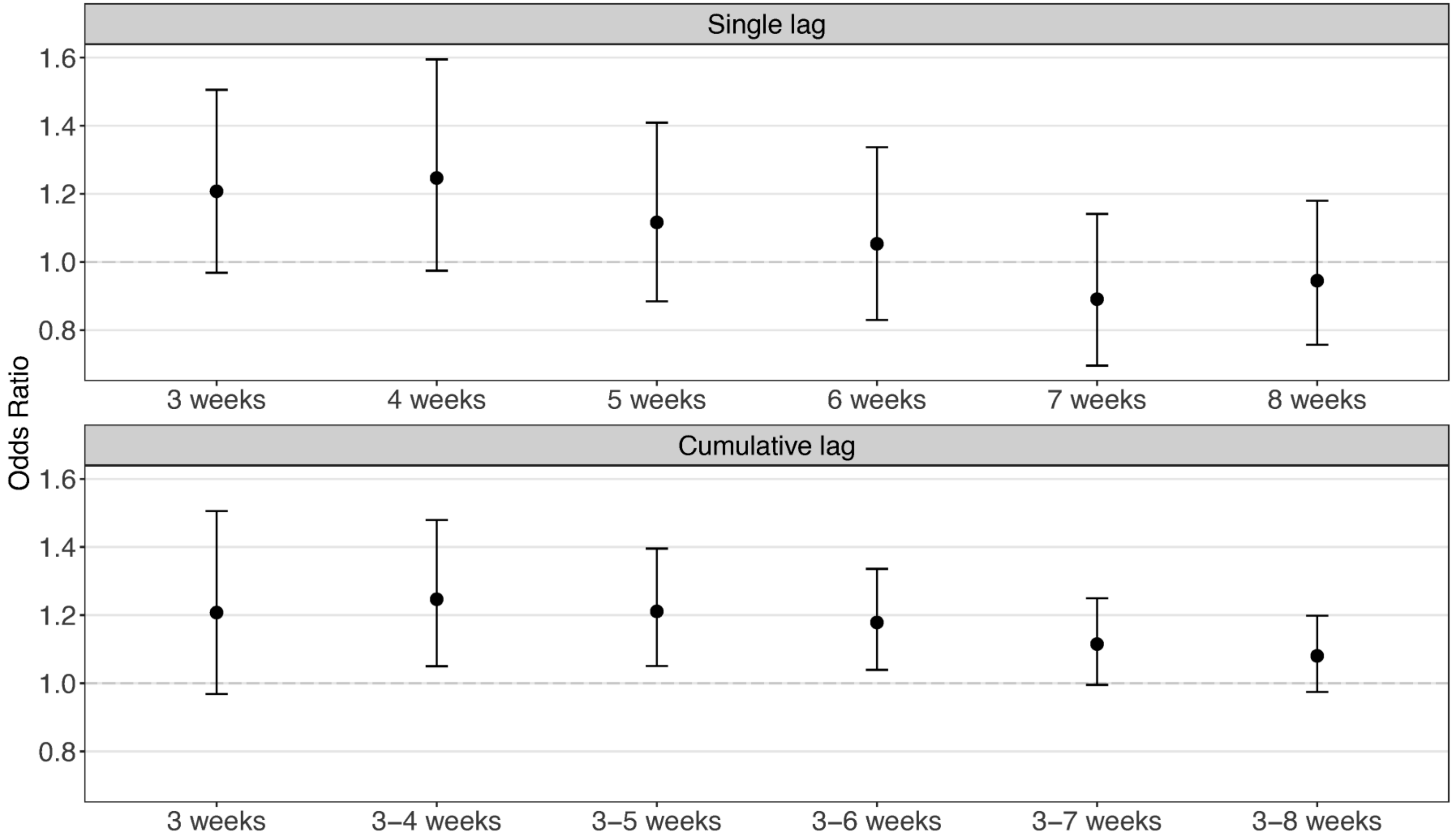

© 2023 Wang P et al. *JAMA Pediatrics.*

eFigure 14. Association Between Exposure to Floods and Risk of Diarrhea Among Children Under 5 Using Multiple Imputation for Missing Values (N = 914 097)

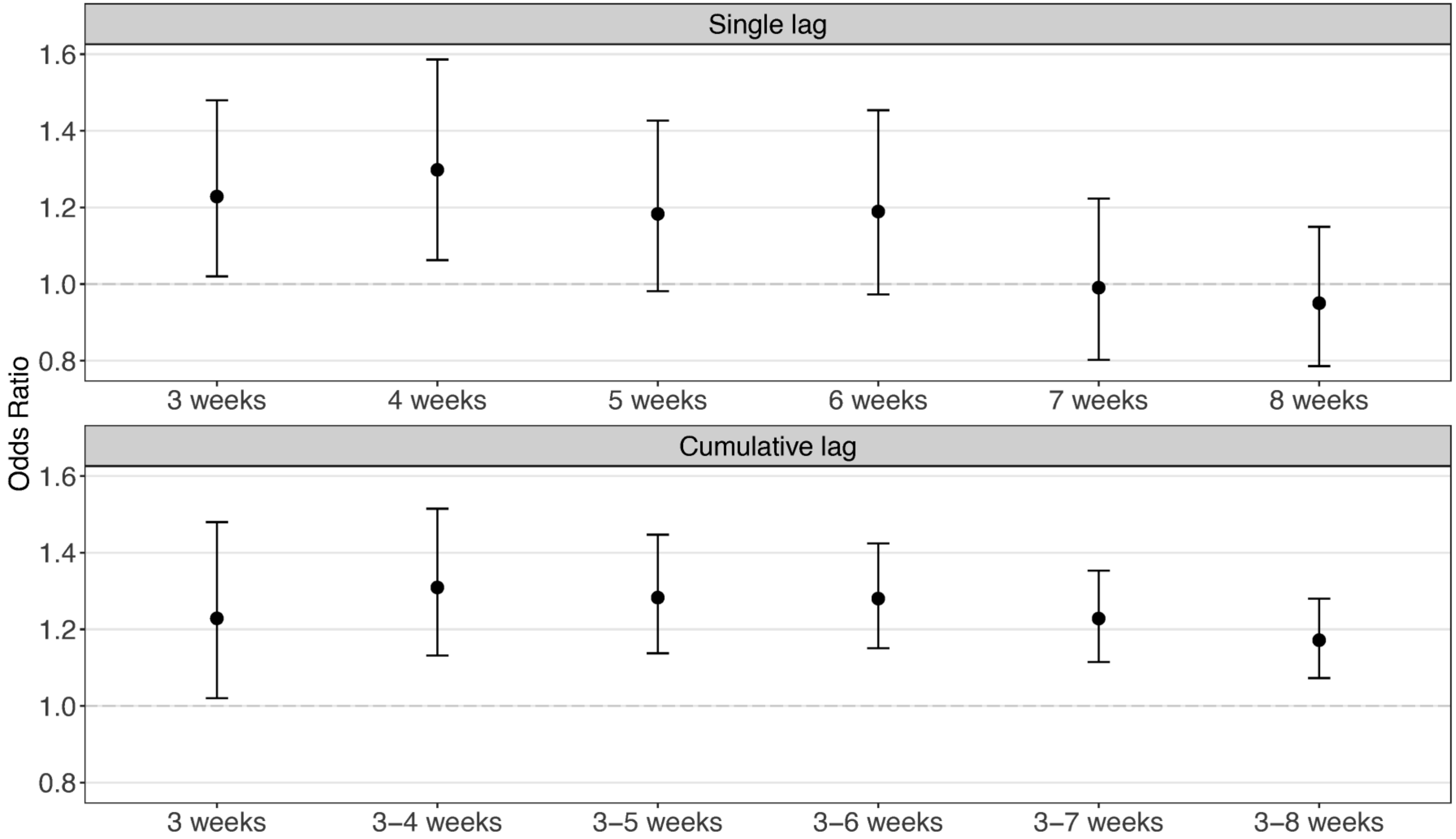

© 2023 Wang P et al. *JAMA Pediatrics.*

eFigure 15. Association Between Exposure to Floods and Risk of Diarrhea Among Children Under 5 Taking Into Account DHS Sampling Weights (N = 639 250)

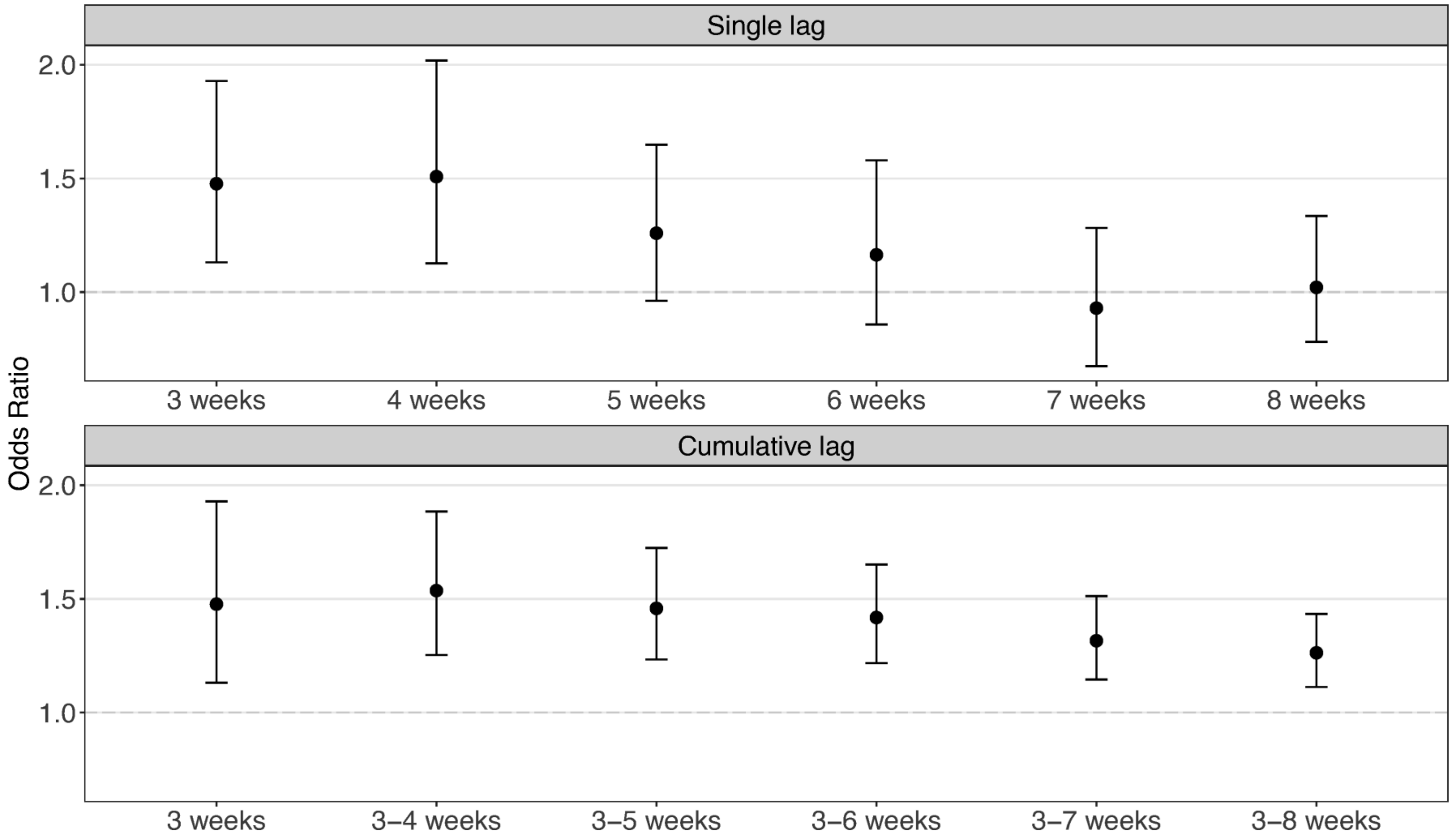

© 2023 Wang P et al. *JAMA Pediatrics.*

eReferences

1. Muñoz-Sabater J, Dutra E, Agustí-Panareda A, et al. ERA5-Land: a state-of-the-art global reanalysis dataset for land applications. *Earth Syst Sci Data.* 2021;13(9):4349-4383.
2. Wang P, Asare E, Pitzer VE, Dubrow R, Chen K. Associations between long-term drought and diarrhea among children under five in low- and middle-income countries. *Nat Commun.* 2022;13(1):3661.
3. Vicente-Serrano SM, Begueria S, Lopez-Moreno JI. A multiscalar drought index sensitive to global warming: the standardized precipitation evapotranspiration index. *J Climate.* 2010;23(7):1696-1718.
4. Federal Office of Meteorology and Climatology MeteoSwiss. Calculation of SPI and SPEI. https://www.meteoswiss.admin.ch/home/climate/swiss-climate-in-detail/climate-indicators/drought-indices/spi-and-spei.html. Accessed Sep 8, 2021.
5. Brakenridge GR. Global Active Archive of Large Flood Events. 2022; http://floodobservatory.colorado.edu/. Accessed 30 November, 2022.

© 2023 Wang P et al. *JAMA Pediatrics.*
