# Supplementary material for: Floods and Diarrhea Risk in Young Children in Low- and Middle-Income Countries
Source: JAMA Pediatr. 2023 Oct 2;177(11):1206–14. doi: 10.1001/jamapediatrics.2023.3964 (PMC10546297; doi:10.1001/jamapediatrics.2023.3964)
Supplement: Supplement 2. — Data Sharing Statemen [file jamapediatr-e233964-s002.pdf]

Data Sharing Statement

Wang. Floods and Diarrhea Risk in Young Children in Low- and Middle-Income Countries. *JAMA Pediatr*. Published October 02, 2023. doi:10.1001/jamapediatrics.2023.3964

Data
Data available: Yes
Data types: Other (please specify)
Additional Information: The source of publicly available datasets is provided with the manuscript.
How to access data: Survey data including diarrhea and socioeconomic data in this study are publicly available upon request from the Demographic and Health Surveys Program (https://dhsprogram.com/). Historical flood data can be obtained from the Dartmouth Flood Observatory (https://floodobservatory.colorado.edu/index.html). Publicly available meteorological records can be downloaded from the fifth-generation ECMWF atmospheric reanalysis of the global climate (ERA5-Land) at https://cds.climate.copernicus.eu/.
When available: With publication

Supporting Documents
Document types: None

Additional Information
Who can access the data: Researchers whose proposed use of the data has been approved.
Types of analyses: For research purpose
Mechanisms of data availability: Without investigator support
